# Progress and Prospect of Organic Electrocatalysts in Lithium−Sulfur Batteries

**DOI:** 10.3389/fchem.2021.703354

**Published:** 2021-07-15

**Authors:** Yangyang Dong, Tingting Li, Dong Cai, Shuo Yang, Xuemei Zhou, Huagui Nie, Zhi Yang

**Affiliations:** ^1^ Key Laboratory of Carbon Materials of Zhejiang Province, College of Chemistry and Materials Engineering, Wenzhou University, Wenzhou, China; ^2^ College of Electrical and Electronic Engineering, Wenzhou University, Wenzhou, China

**Keywords:** lithium−sulfur battery, organic electrocatalyst, sulfur conversion, structure–activity relationship, reaction kinetics

## Abstract

Lithium−sulfur (Li−S) batteries featured by ultra-high energy density and cost-efficiency are considered the most promising candidate for the next-generation energy storage system. However, their pragmatic applications confront several non-negligible drawbacks that mainly originate from the reaction and transformation of sulfur intermediates. Grasping and catalyzing these sulfur species motivated the research topics in this field. In this regard, carbon dopants with metal/metal-free atoms together with transition–metal complex, as traditional lithium polysulfide (LiPS) propellers, exhibited significant electrochemical performance promotions. Nevertheless, only the surface atoms of these host-accelerators can possibly be used as active sites. In sharp contrast, organic materials with a tunable structure and composition can be dispersed as individual molecules on the surface of substrates that may be more efficient electrocatalysts. The well-defined molecular structures also contribute to elucidate the involved surface-binding mechanisms. Inspired by these perceptions, organic electrocatalysts have achieved a great progress in recent decades. This review focuses on the organic electrocatalysts used in each part of Li−S batteries and discusses the structure–activity relationship between the introduced organic molecules and LiPSs. Ultimately, the future developments and prospects of organic electrocatalysts in Li−S batteries are also discussed.

## Introduction

**SCHEME 1:**
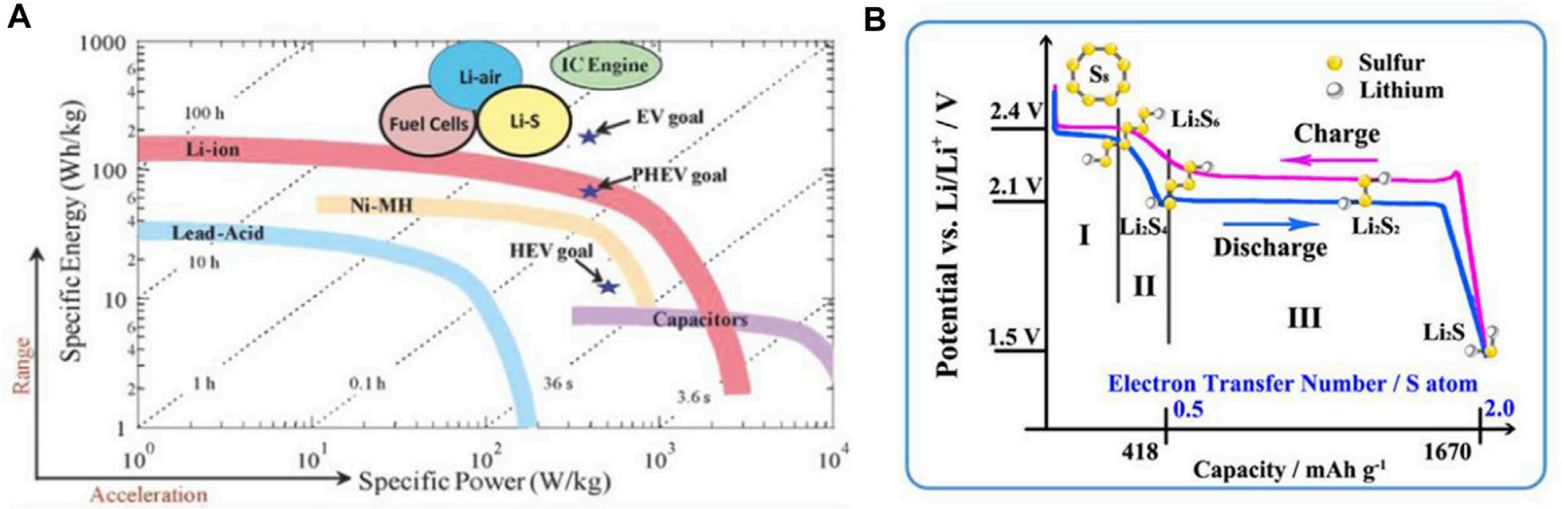
**(A)** Ragone plots of typical energy storage systems. **(A)** Figure reproduced from the data in Rosenman et al. (2015). **(B)** The galvanostatic charge–discharge profiles of a typical Li−S battery and the corresponding products at each voltage stage. **(B)** Figure reproduced from data in Liang et al. (2016).

The pursuit for high-energy-density secondary batteries is still in progress and has never been held up due to the painfully impaired by gross abuse of fossil fuels (Peng et al., 2017). Beyond traditional lithium-ion batteries (LIBs), new emerging battery systems such as lithium/sodium-oxygen (Li/Na−O_2_), lithium/sodium-sulfur (Li/Na-S), and zinc-oxygen/sulfur (Zn-O_2_/S) with ultra-high expected values are chasing the “Holy Grail” (Zhang et al., 2017). In particular, featured by a theoretical specific capacity of 1,675 mAh g^−1^ and an energy density of 2,600 Wh kg^−1^, the Li−S system has become one of the leaders (Scheme 1A) (Rosenman et al., 2015; He and Manthiram, 2019). Nevertheless, the development of Li−S batteries has never been a smooth sailing. The insulation of bulk sulfur (S_8_) is the priority to consider that will hinder the electron transfer (5.0 × 10^−30^ S cm^−1^), leading to sluggish sulfur redox reactions and low sulfur utilizations (Li C. et al., 2017). If, fortunately, the S_8_ molecules received electrons, the S-S bond would be broken to form long-chain lithium polysulfide (LiPS) molecules (Li_2_S_n_, *n* = 8, 7, 6, 5, 4) (Hong et al., 2020). The as-obtained LiPSs are soluble in conventional ether electrolyte and can diffuse to lithium (Li) anode (as called “shuttle effect”) to generate solid Li_2_S_2_/Li_2_S precipitations, resulting in the continuous consumption of S and low Coulombic efficiency (Scheme 1B) (Pope and Aksay, 2015) (Liang et al., 2016). Moreover, in this scenario, the mass density variations between S (2.07 g cm^−3^) and Li_2_S (1.66 g cm^−3^) cause large volume changes up to ∼80%, deteriorating the rationally designed configurations of the cathode (Zhang et al., 2020a). Last, but not the least, the lithium ions (Li^+^), during the repeated charge/discharge process, are inclined to unevenly deposit on the surface of Li anode, which induce the formation of Li dendrites and raise the potential security concerns (Guo J. et al., 2021). Overall, the above four aspects constitute the main bottlenecks of Li−S batteries since their first proposal.

The remedy for the problems, by general consent, lies in the hands of material innovation. In the early stage, scientists mainly focused on advanced materials for physical encapsulation (Peng and Zhang, 2015) and chemical adsorption (Eftekhari and Kim, 2017) of LiPSs. To physically encapsulate LiPSs, various carbon materials have been developed. However, their nonpolar surface cannot effectively block the shuttle effects of polar LiPSs (Sun Z. et al., 2020). Under this condition, the solution is to improve the polarity of carbonaceous materials, creating strong chemisorption effects. For instance, the heteroatoms (N, O, S, P, etc.) as exposed active sites on carbon materials can adsorb and enrich LiPSs at the electrochemical interfaces through the formation of chemical bonds (Wang J. et al., 2020). The high concentration of LiPSs accumulated at the highly active interfaces can speed up the conversion reactions and enhance the reaction kinetics. Thus, the chemical adsorption of LiPSs favors uniform distribution of S and insoluble Li_2_S_2_/Li_2_S on hosts, ensuring a strong electrical contact with conductive substrates and efficiently reducing the dissolved LiPSs (Hou et al., 2016). However, the effects by chemical adsorption and physical barrier require large amounts of porous carbon materials which may consume flooded electrolyte, thereby remarkably reducing the output energy density. In addition, chemical adsorption sites cannot be precisely controlled by simply doping methods. Consequently, it also calls for dynamic regulations of reaction procedures to solve these problems.

**SCHEME 2:**
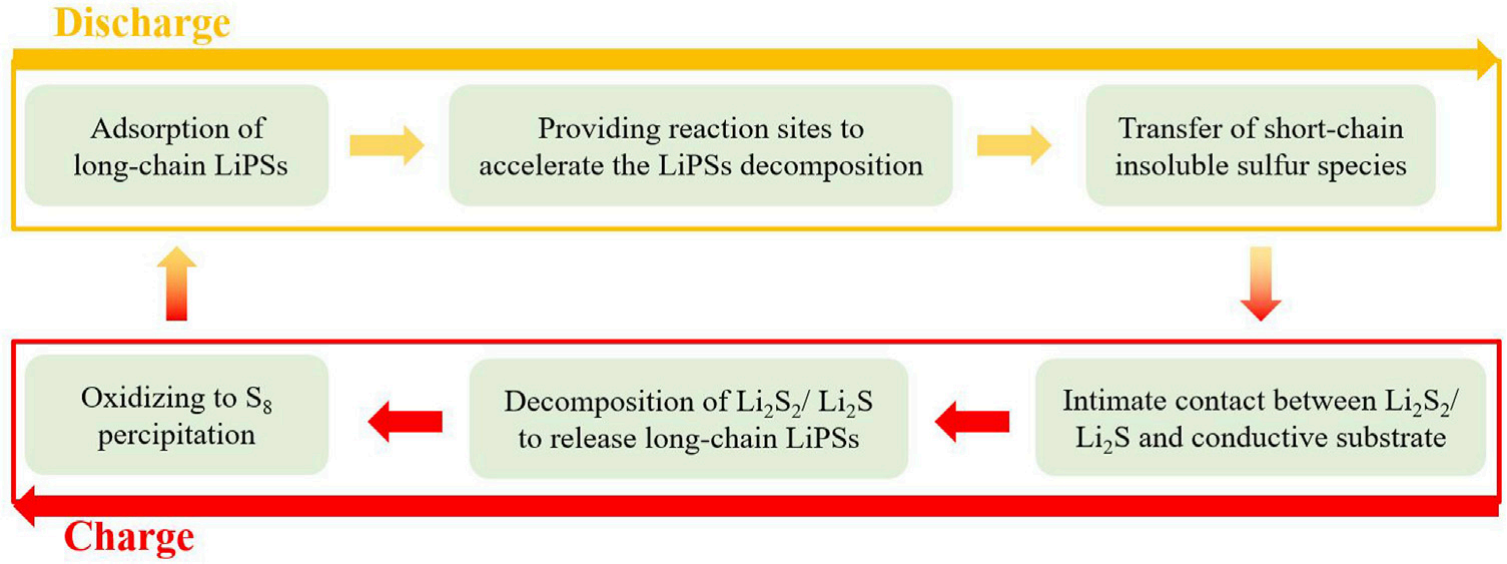
The dynamic catalytic cycle of sulfur conversion in Li−S batteries.

In a Li–S battery, the sulfur redox reactions involve a multistep procedure with two electron transfer (1/8S_8_ + 2Li^+^ + 2e^−^ ↔ Li_2_S) and the main capacity can be ascribed to the conversion of long-chain LiPSs to their short-chain deformations (Seh et al., 2016). Given the slow reaction kinetics for the reduction of short-chain LiPSs to Li_2_S_2_/Li_2_S, the formation rate of long-chain LiPSs is greater than their consumption, causing a large amount of soluble LiPSs accumulated on the sulfur cathode. In this process, owing to a solid–solid reaction, the conversion of Li_2_S_2_ to Li_2_S is the rate-controlling step (Li G. et al., 2018). As a result, apart from the above-mentioned adsorptions and confinements, the catalysis toward LiPSs should also be carefully checked. Catalytic sulfur conversion, as an efficient tactic, is employed to reduce the concentration and retention period of residual LiPSs in electrolyte and the amount of insoluble S and Li_2_S_2_/Li_2_S on the surface of cathode (Huang et al., 2020). Designing catalysts in Li−S systems can accelerate the charge transfer, reduce the voltage hysteresis, and thus improve the rate capability, as well as the sulfur utilization. Noted that, in traditional catalytic systems, catalysis mainly includes three processes: adsorption, conversion, and desorption (Zhou H.-J. et al., 2020). In this system, adsorption is also regarded as a prerequisite step for catalysis (Hong et al., 2020). The main goal of catalytic materials is to improve the sulfur conversion efficiency, namely, the absolute conversion amounts and rates. Therefore, in a broad sense, as long as the material can promote the utilization of sulfur and accelerate the reaction rate, it is called a catalyst. For a certain discharge/charge process, catalysis of sulfur conversion includes six main dynamic processes: 1) absorb soluble long-chain LiPSs near the catalysts; 2) the catalysts provide reaction sites to accelerate the LiPS decomposition; 3) the transfer of short-chain insoluble sulfur species on the surface of catalysts; 4) an intimate contact between transferred Li_2_S_2_/Li_2_S and conductive substrates; 5) decomposition of Li_2_S_2_/Li_2_S to release long-chain LiPSs; and 6) the final oxidization of long-chain LiPSs to initial reactants (S_8_). Process 1–3) occurs during the sulfur reduction (discharge) stage, and 4–6) represent the relevant oxidation (charge) stage. These dynamic processes can be described as Scheme 2. Hence, the desired new functional materials in Li−S batteries should take advantage of the above-mentioned adsorption and catalysis.

**SCHEME 3:**
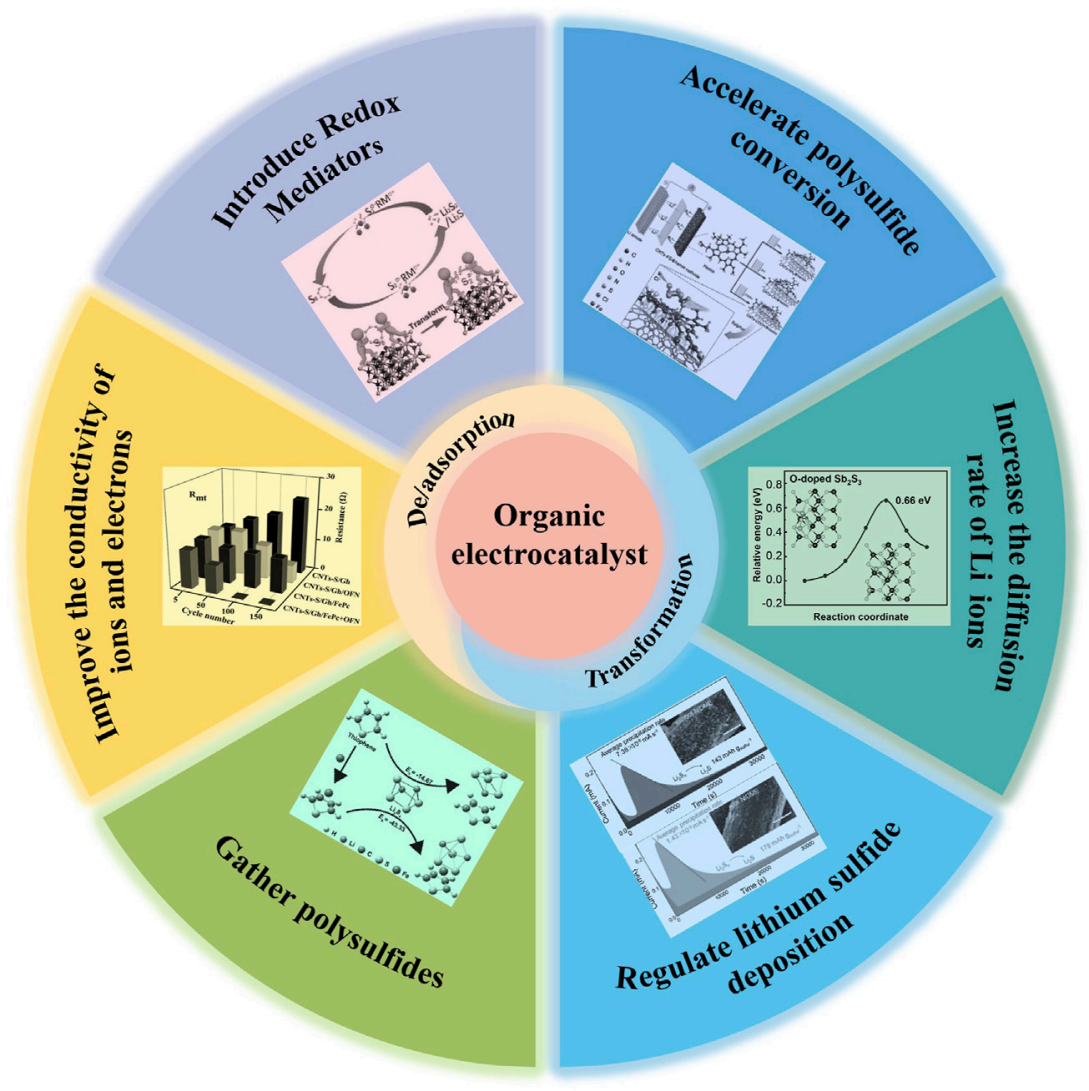
Functionalities and effects of organic electrocatalyst in Li−S systems.

Organic materials with a tunable structure and abundant composition become more efficient electrocatalysts for propelling sulfur conversions that can be dispersed as individual molecules on the surface of substrates (Qu et al., 2020). The well-defined molecular structures also contribute to elucidate the involved surface-binding mechanisms. Inspired by these perceptions, organic electrocatalysts have achieved great progresses in Li−S batteries within recent few decades, which may improve the conduction of electrons/ions, shackling LiPSs, increasing the diffusion rate of Li ions, and regulating the deposition of Li_2_S_2_/Li_2_S. However, there is a scarce of comprehensive summary and in-depth analysis for these reports until now. For those concerns, this review summarizes recent designs of organic electrocatalysts in each ingredient of a Li−S battery, as shown in Scheme 3. The adsorption and conversion of sulfur species by organic electrocatalyst will be discussed accordingly, accompanied by future perspectives to improve their electrochemical performances.

## Organic Electrocatalysts in Sulfur Cathode

The role of organic electrocatalysts in a Li−S battery includes: 1) chemically bind LiPSs through active sites to inhibit the shuttle effect (Li N. et al., 2021); 2) accelerate the redox reactions of LiPSs (Wang C. et al., 2020); 3) improve the utilization of active materials by regulating the nucleation and growth kinetics of Li_2_S (Yang X. et al., 2019). The introduction of organic electrocatalytic materials on the sulfur cathode is considered to be a feasible solution for addressing problems in Li−S batteries. In this section, we will introduce their applications from the following three parts. In the first section, organic electrocatalytic materials for sulfur hosting substrates are explicated. Subsequently, we discuss organic electrocatalytic additives in sulfur cathode. At last, organic electrocatalysts as new binders are summed up and summarized.

### Organic Electrocatalysts for Sulfur Hosts

The sulfur host, as the modifier additive, was introduced to be a robust framework for constructing electron/Li^+^ conduction channels in Li–S batteries so as to alleviate large volume expansions (Zhao et al., 2019), deposit insoluble Li_2_S_2_/Li_2_S sediments (Zhang S. et al., 2021), and limit the migration and permeation of LiPSs into the electrolyte (Yan et al., 2019). Considering the existence of sulfur species, the host plays pivotal roles in regulating the adsorption and transformation of LiPSs. Nanostructured carbon materials have been considered good sulfur hosts (Zeng et al., 2014; Li W. et al., 2016; Patel et al., 2017; Zhang Y. et al., 2018; Zhang J. et al., 2018; Zhang et al., 2019b; Zheng et al., 2019; Chen et al., 2019). To a certain extent, they can physically confine LiPSs in pore structures, inhibiting their dissolution in electrolyte and improving the electrochemical performance (Liang et al., 2016). However, their weak interactions with polar LiPSs lead to their separations in a long run, which limits practical applications (Xie et al., 2019). Therefore, polar metal-based compounds have been introduced. Compared with carbon materials, they take strong chemisorption and catalysis on LiPSs, and can efficiently prohibit the shuttle effect (Wang et al., 2018). However, there may be two remarkable disadvantages, that is, metal-based compounds possess limited active sites on the surface and most of these sites are blocked in the bulk. As a result, the catalysis cannot be fully exerted. On the other side, the high mass density will compromise the high theoretical energy density of this system. Demobilizing the sulfur by attaching it on the organic backbone has been proved to be an effective way for improving the overall performance of Li−S batteries (Shadike et al., 2021). Therefore, organic electrocatalysts with fully exposed active sites and light-weight superiority have attracted extensive research interests. Herein, we will discuss and summarize typical electrocatalytic sulfur hosts by organic materials.

#### Metal Organic Frameworks

Metal atoms in metal organic frameworks (MOFs) are regarded as Lewis acidic sites and nonmetallic anions in ligands as Lewis basic sites (Lu et al., 2018) which can interact with S_n_^2-^ anions and Li^+^ cations in LiPSs, respectively. By synergistic effects of these active sites, it is expected to effectively solve the shuttle phenomenon of LiPSs. As a typical case, zeolite imidazole ester frameworks (ZIFs) combine the dual advantages of MOFs (high porosity and large specific surface area) and zeolite (high stability), showing application potential in many aspects (Li S. S. et al., 2018; Wei et al., 2021). Among the aspects, metallic nickel atoms with variable oxidation states are widely used in the field of electrocatalysis (Yang H. B. et al., 2018). Based on these, Yang’s group designed a three-dimensional (3D) heterogeneous sulfur host (Ni-ZIF-8@CC) by *in-situ* depositing nickel-doped ZIF-8 on carbon clothes (Yang Y. et al., 2018). The following experiments demonstrate the strong chemical interactions of Ni-ZIF-8@CC with LiPSs through Ni-S and N-Li bonding, inhibiting the shuttle effect of LiPSs. The Li^+^ diffusion characteristics at different scan rates indicate that the Ni-ZIF-8@CC/S cathode possesses faster Li^+^ diffusion capabilities. In subsequent charge/discharge tests, the Ni-ZIF-8@CC/S shows a much smaller voltage hysteresis of 0.16 V, giving an evidence of the faster oxidation–reduction reaction kinetics. The as-prepared Li−S batteries can deliver a high initial discharge capacity of 6.04 mAh cm^−2^ under a sulfur loading of 5.5 mg cm^−2^. More impressively, it maintains 5.3 mAh cm^−2^ after 100 cycles. The other typical case is the regulation of organic ligands in MOFs. A large number of researchers have noticed the positive effects of 2,4,6-tris(3,5-dicarboxylic acid aniline)-1,3,5-triazine (H_6_TDPAT) in Li–S batteries that derive from melamine (Gang et al., 2021). It is saturated with N active sites that can chemically bond with Li^+^. By similar Lewis acidic–basic interactions, Hong et al. reported the introduction of dual-functional MOF cages with central cooper sites (Cu-MOFs) for capturing sulfur species and catalyzing their conversion (Hong et al., 2018). The electrochemical tests prove that the optimized Cu-TDPAT MOFs enables good discharge capacity and cycling performance. The discharge capacity remains 745 mAh g^−1^ after 500 cycles at a current density of 1 C.

Although the research on chemically binding LiPSs through Lewis acidic–basic mechanisms has been well developed, it is evident that the low electron/ion conductivity of traditional MOF severely limits the electrochemical kinetics of sulfur conversion, resulting in its low utilization. It is urgent to develop high conductive MOF materials. The first choice is to search for proper anionic ligands. A great number of groups have reported that highly symmetrical planar structured ligand of hexamercaptobenzene (BHT) with rich chemical coordinations could be employed to construct a two-dimensional (2D) copper-based MOF (Cu-BHT) (Wu et al., 2020). The unique composition and clathrate crystal structure enable Cu-BHT a high electronic conductivity of up to 1580 S cm^−1^ at room temperature (Huang X. et al., 2018). Li et al. reported that the Cu-BHT-based sulfur host shows not only a strong chemical interaction with LiPSs, but also attains high electrochemical reaction kinetics (Li F. et al., 2018). Their good affinities toward LiPSs promote uniform depositions of Li_2_S. All of the above factors are in favor of catalyzing the conversion of sulfur species. Delightingly, 2D MOFs with a π-π conjugated structure and excellent conductivity, such as Ni(BHT) and Cu(HITP), have proliferated over the last few years that may lead further research in this direction. The other strategy is the coating of conductive polymers on pristine MOFs. A classic case is the conductive polypyrrole (Shi et al., 2014) that possesses a high conductivity of up to 10–100 S cm^−1^, superior tensile strength, and good electrochemical redox reversibility. Guided by this dominated thinking, Geng’s group achieved conductivity improvement by coating polypyrrole on the outer layer of sulfur-infiltrated hollow ZIF-67 hosts (Geng et al., 2019). This organic coating can effectively buffer the volume expansion of sulfur species. The experimental results demonstrated that this nonconductive MOF@conductive polymer composite can deliver much more stable cycle performance. Conducting electrons/ions through the polymer is helpful to the catalysis and activity of MOFs. It provides a new synthetic method for the preparation of high-conductive MOF-based sulfur hosts.

**FIGURE 1:**
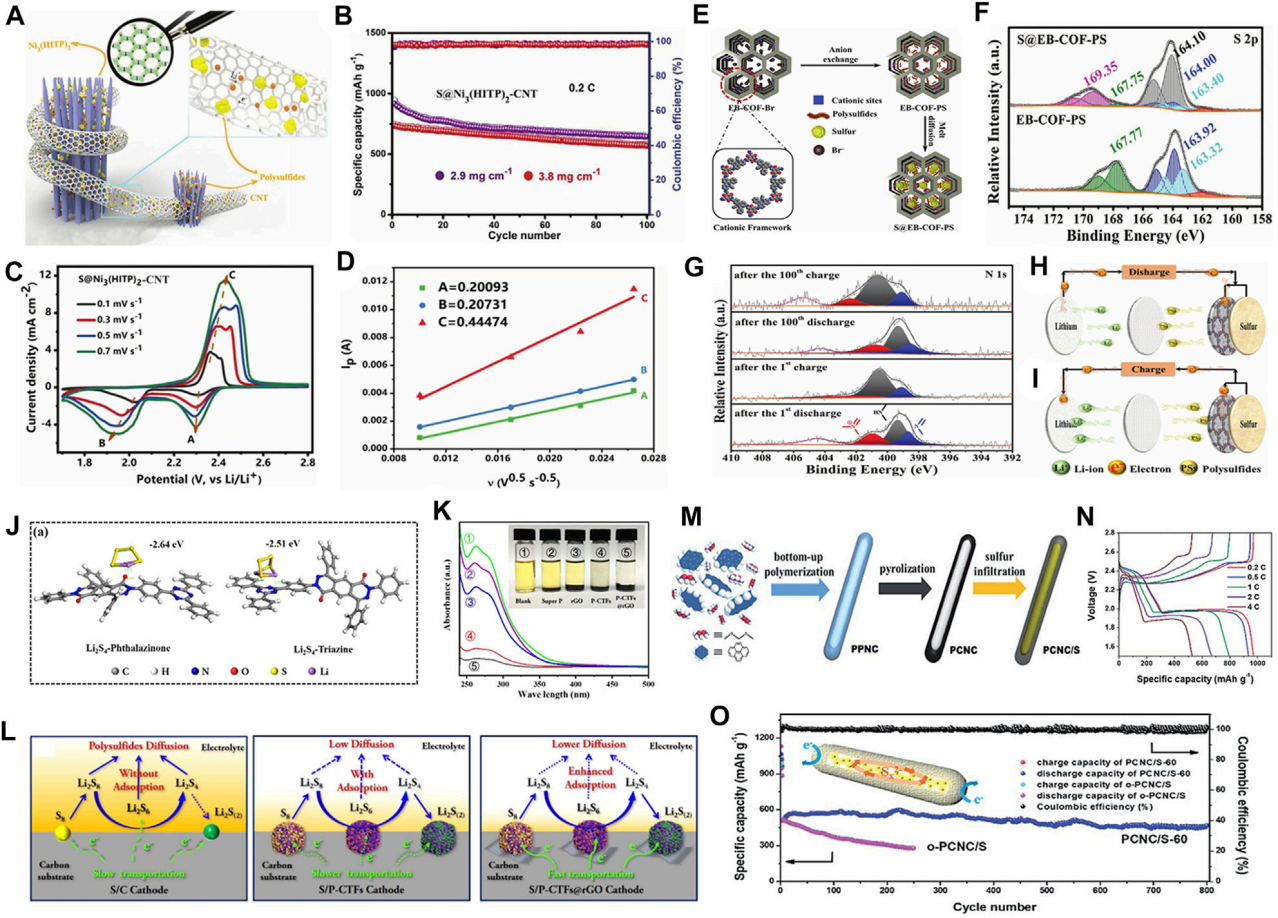
Illustrations of organic electrocatalysts for sulfur hosts. **(A)** Synergistic effects of Ni_3_(HITP)_2_ and CNT on Li−S batteries. The enlarged image is the crystalline of Ni_3_(HITP)_2_. **(B)** Cycling performance of S@Ni_3_(HITP)_2_-CNT cathodes at sulfur loadings of 2.9 and 3.8 mg cm^−2^ under 0.2 C. **(C)** CV profiles of S@Ni_3_(HITP)_2_-CNT at various scan rates. **(D)** The fitted Li^+^ diffusion coefficient of S@Ni_3_(HITP)_2_-CNT at different redox peaks which indicates the improvement of dynamic performance by synergistic effects of Ni_3_(HITP)_2_ and CNTs. **(A–D)** Figure reproduced from the data in Cai et al. (2019). **(E)** A schematic diagram of the synthesis of S@EB-COF-PS. **(F)** S 2p XPS spectra of EB-COF-PS and S@EB-COF-PS. **(G)** N 1s XPS spectra of S@EB-COF-PS after 1 and 100 cycles of charge/discharge. **(H–I)** Illustrations of the S@EB-COF-PS battery during discharge/charge processes. **(E–I)** Figure reproduced from the data in Zeng et al. (2017). **(J)** The optimized structure of phthalazinone and triazine functioned groups combined with Li_2_S_4_. **(K)** Ultraviolet-visible absorption spectra and optical photos of Super P, reduced graphene oxide (rGO), P-CTFs, and P-CTFs@rGO immersed in Li_2_S_6_ solutions. **(L)** A schematic diagram of the discharge process of S/C, S/P-CTFs, S/P-CTFs@rGO cathodes. **(J–L)** Figure reproduced from the data in Troschke et al. (2020). **(M)** The synthetic processes of PPNC, PCNC, and PCNC/S. **(N)** Charge and discharge curves of PCNC/S-60 cathode at different current densities. **(O)** Cycling performance of PCNC/S-60 and o-PCNC/S cathodes at 4 C for 800 cycles. The inset is a schematic diagram of the capsule structure. **(M–O)** Figure reproduced from the data in Xiao et al. (2019).

To further improve the conductivity of MOF-based sulfur hosts, researchers have launched new attempts. They combined the above-mentioned two strategies to make the composite with both highly conductive MOFs and conductive layer coatings. The conductivity of Ni_3_(2,3,6,7,10,11-hexaiminotriphenylene)_2_ [Ni_3_(HITP)_2_], discovered by Mircea Dincă, reaches a record value of 5000 S m^−1^, even exceeding most activated carbons and holey graphite (∼1,000 S m^−1^). This kind of MOFs has functioned as key materials in semiconducting electronics, electrocatalysts, and energy storage systems (Vlad and Balducci, 2017). Cai et al. adopted a hydrothermal method to synthesize this 2D layered Ni_3_(HITP)_2_ for Li−S sulfur hosts (Cai et al., 2019). Besides, carbon nanotubes (CNTs) additives were used to realize the effective combination of short-range conductive Ni_3_(HITP)_2_ and long-range conductive CNTs, which establish a fast ion/electron transport matrix [S@Ni_3_(HITP)_2_-CNT] (Figure 1A). Cyclic voltammetry (CV) tests showed that the S@Ni_3_(HITP)_2_-CNT displays higher peak current densities at points of redox reactions. A new oxidation peak appeared at around 2.42 V, indicating that the oxidation process of LiPSs was more efficient and more thorough. Consequently, the S@Ni_3_(HITP)_2_-CNT provides a high initial discharge capacity of 1,302.9 mAh g^−1^ at a current density of 0.2 C and retains 848.9 mAh g^−1^ after 100 cycles. Even at a high sulfur loading of 2.9 and 3.8 mg cm^−2^, the S@Ni_3_(HITP)_2_-CNT cathode can still maintain 643 and 568 mAh g^−1^ after 100 cycles (Figure 1B), showing good practical possibilities. To explore the reaction kinetics, CV tests at different scan rates were carried out (Figure 1C). The scan rates and the peak currents can be linearly correlated (Figure 1D). Compared to control groups, it possesses a higher integral area at various scan rates that means a higher Li^+^ diffusion coefficient, suggesting promoted LiPSs conversion kinetics. On the basis of this research, the synergistic promotion of multi-components on the cathode side would be realized. Baumann et al. proposed a synergistic effect of highly porous Zr-based MOFs-808, which is easy to synthesize and rich in metal Zr active sites, with conductive graphene and a surfactant additive (ethyl cellulose) (Baumann et al., 2020). It reveals that soluble LiPSs can be severely restricted in the cathode. Among them, graphene nanosheets can increase the conductivity and guide the electron/ion transfer. Meanwhile, the ethyl cellulose additives pull graphene and active sulfur particles into intimate contact that may obtain high volumetric energy density. Decomposing various parts of the catalytic conversion processes, each component that reaches to its best will ultimately contribute to the overall performance improvements. On the basis of the above, the prepared MOFs-808-based cathode presents much lower polarization voltage and better cycle stability.

#### Covalent Organic Frameworks

Covalent organic frameworks (COFs) are 2D-layered crystalline structures with designable π-electron frameworks and highly ordered topological forms that are constructed by covalent bonding of light elements such as C, O, N, B, etc. and then undergo a reversible polymerization process. The unique composition and structure endow COFs with high porosity, large specific surface area, low mass density, ordered and open nano-channels, and high thermal/chemical stability, presenting potential applications in fields such as heterogeneous catalysis, energy storage, optoelectronic, and sensing (Li J. et al., 2020). Meng’ group proposed a 2D pyrene-based COF (Py-COF) for organic electrocatalysts (Meng et al., 2018). In this scenario, they found a noticeable improvement by using Py-COF, that is, an elevated Li^+^ transport capability. After cycling for 550 cycles, the Py-COF-based sulfur host can still deliver a high discharge capacity of 481.2 mAh g^−1^ at a current density of 5.0 C together with a mean decay rate of 0.048% per cycle. However, pours of other reports suggest that traditional COFs should not be directly used as the sulfur matrixes due to the lack of active centers for anchoring LiPSs.

In recent few years, researches have focused on introducing polar functional groups into COFs to chemically bind with LiPSs by forming such nonmetallic-Li bonds for further enhancing their interactions (Ghazi et al., 2016). Lu et al. devised a 2D COF (COF-ETTA-ETTCA) with uniform micropores and an extended π-conjugated structure through the condensation reaction of aldehydes and amines (Lu et al., 2020). The experiments and theoretical calculations make a consensus of accelerated electron transports by these conjugate structures. In addition, X-ray photoelectron spectroscopy (XPS) tests give a solid evidence that the doping of N atoms can interact with Li^+^ in LiPSs, and thus improves the wettability, surface polarity, and adsorption capacity of COFs toward LiPSs. Based on this structure–activity relationship, the as-prepared COF-based sulfur cathode maintains a stable electrochemical performance for more than 500 cycles.

Quaternary ammonium salt is similar to inorganic salt that is soluble in water and can conduct electricity. It is usually used as phase transfer catalysts in chemical reactions. In a recent work, after carefully analyzing the effects by introducing quaternary ammonium salts in a certain COF (EB-COF-PS), Zeng et al. concluded that the cationic active sites can strengthen the anchoring effect of LiPSs (Figure 1E) (Zeng et al., 2017). To study detailed interactions between them, high-resolution S 2p XPS spectroscopy was carried out (Figure 1F). The characteristic peaks at 164.00 and 163.40 eV are attributed to terminal sulfur (S_T_^−1^) and bridge sulfur (S_B_^0^), respectively. The new peak at 164.10 eV can be assigned to elemental sulfur. Compared to control groups, these obviously shifted peaks imply effective interactions between cationic sites and polysulfide anions. According to further explorations, they speculated the mechanisms behind: the cationic sites can accept electrons and transport them to the LiPSs during the discharge process (Figure 1H), thereby promoting the decomposition of LiPSs; upon charging (Figure 1I), the cationic sites receive electrons from the LiPSs, and send them to current collectors for promoting the oxidation of LiPSs. As a result, the S@EB-COF-PS-based Li−S battery presents an amazing specific discharge capacity of 468 mAh g^−1^ after 300 cycles at a current density of 4.0 C, showing significantly improved dynamic performance. This work highlights the construction of cationic sites in COFs.

#### Covalent Triazine-Based Organic Frameworks

With the depth of research, scientists show solicitude for the study of covalent triazine-based organic frameworks (CTFs). Similar to COFs, CTFs are characterized by their large surface area, high stability, flexible synthesis strategy, and multi-functionality (Troschke et al., 2020). Through summarizing related literatures, it is found that covalent triazinyl can significantly improve the anchoring capability of LiPSs by doping heteroatoms. There are seven electrons in the outermost electronic structure of a fluorine atom that is easy to obtain an electron to reach a stable state. Therefore, the fluorine atom has a relatively large electronegativity and can chemically bond with Li^+^ in LiPSs. A pioneer work was reported by Xu and colleagues that fluorine-based functional groups (FCTF) were grafted on porous triazinyl backbones that could effectively inhibit the dissolution of LiPSs and accelerate their conversion (Xu et al., 2017). The composite cathode exhibits a high discharge capacity of 1,296 mAh g^−1^ at a current density of 0.1 C, and maintains 833 mAh g^−1^ after 150 cycles under 0.5 C, showing good cycling performance. The influence of heteroatom doping on the LiPSs conversion reaction was further explored by Jian et al. that they have prepared various N, O codoped CTFs (NO-CTF-1 and NO-CTF-2) for Li–S batteries (Zhang T. et al., 2021) and discovered at least two advantages by this devise: 1) the N and O heteroatoms in NO-CTFs can provide strong Lewis acidic–basic interactions with Li atoms in LiPSs; 2) the unique pore structure and efficient catalytic effects propel the rapid Li_2_S nucleation reactions, accelerating the conversion of LiPSs.

Although heteroatom dopings in CTFs strengthened the combination with LiPSs, the conductivity needs further improvement and thoughts turn first to light-weight carbon matrixes. Troschke et al. constructed a porous CTF with phthalazine *in-situ* on rGO sheets (P-CTFs@rGO) where the rGO acted as both conductive matrixes and Li_2_S/Li_2_S_2_ precipitation sites (Troschke et al., 2020). After modification, a large number of active groups such as phthalazinone and triazine were presented in these polar hosts. The Density Function Theory (DFT) calculations (Figure 1J) confirmed strong adsorption capabilities of introduced phthalazinone and triazine functional sites toward LiPSs, as proved by ultraviolet-visible (UV-vis) absorption spectra and visualization of adsorption experiments (Figure 1K). Compared with other samples, the color of Li_2_S_6_ solution in the presence of P-CTFs@rGO decays fastest, showing weakest absorption signal in UV-vis spectra. In CV profiles, only one oxidation peak was observed in S/C and S/P-CTFs cathode (control groups) which can be ascribed to the slow oxidation reactions. In contrast two distinct reduction/oxidation peaks were found in the S/P-CTFs@rGO cathode with positive/negative shifts, respectively, proving the improvement of sulfur redox reaction kinetics. In the following electrochemical impedance spectroscopy (EIS) tests, the ohmic resistance (Ro) of the S/P-CTF cathode is comparable with that of the S/C, indicating limited conductivity of P-CTFs. The shortness can be solved effectively by the introduction of rGO. As a result, the Ro value of the S/P-CTF@rGO reduced significantly. In the long-cycle testing at 0.5 C, the discharge capacity of the as-prepared S/P-CTFs@rGO cathode can maintain at 920 mAh g^−1^ after 500 cycles (Figure 1l). These results demonstrated the feasibility of this strategy that combines the strong chemical interactions of CTF-based polar functional groups and the high conductivity of rGO to chemically adsorb LiPSs and catalyze their conversion.

#### Conductive Polymers

As indicated by discussions, a common shortcoming of the above-mentioned MOFs/COFs/CTFs is the limited conductivity. While introducing enough active sites, the rapid transfer of electrons and ions in electrochemical reactions should also be ensured to realize the efficient conversion of sulfur. Conductive polymer-based organic electrocatalytic materials hold these two advantages simultaneously. Besides, the tunable specific surface area, adjustable functional groups, and light specific gravity are also fascinating for a sulfur host (Gao et al., 2020). Even after a carbonization treatment, the active sites still exist on the surface while the electrical conductivity could be greatly improved. Through a series of approaches, it is expected to achieve overall improvements of a Li–S battery. As a specific application case, Xiao’s group reported that porous carbon nanocapsules (PCNC) derived from one-dimensional polypyrene with closed configuration and high conductivity are ideal sulfur hosts for Li−S batteries (Figure 1M) (Xiao et al., 2019). This polypyrene with a well cross-linked framework is rich in porosity and large internal void space that will be preserved after pyrolysis. The galvanostatic charge–discharge tests under different current densities are presented in Figure 1N which show that the PCNC cathode still exhibits a stable dual-platform discharge behavior, even at a high current density of 4 C. When the sulfur content rises up to 76.4 wt%, it can still hold a high discharge capacity and express a good cycling stability. After 800 cycles, the mean capacity decay rate is as low as 0.011% per cycle (Figure 1O). The significant improvements have been unfolded by employing a conductive polymer or its derivatives in many other literatures; however, the complex structural configuration and lack of awareness of interactions between various functional sites and LiPSs restrict their further development.

In general, the main problems of MOF-, COF-, and CTF-based organic electrocatalytic materials are the low conductivity and the weak binding capability with LiPSs. In current research, scientists have proposed a variety of solutions. The insufficient interactions with LiPSs can be settled by decorating with polar functional groups. The low conductivity may be ameliorated by possible solutions: 1) adjusting the structure and composition of the organic ligands; 2) coating a conductive polymer on their surface; 3) combining with conductive carbon materials; and 4) pyrolysis treatments. Besides, more characterization techniques are also required to further get insights into the reaction mechanisms and structure–activity relationship behind.

### Organic Electrocatalytic Additives

When organic electrocatalysts are used as additives in a cathode, it should take careful consideration of their poor conductivity and agglomeration (Ji and Nazar, 2010). More lethal is the soluble of organic electrocatalytic additives in an electrolyte. As a result, the structural integrity of the cathode may be destroyed. Besides, the dissolved organic electrocatalysts in the electrolyte would hinder the ion transport and even react with Li. Large numbers of studies have shown that the optimizations can be taken from the following three aspects: 1) the uniform distribution of sulfur on its hosts; 2) effective inhibition of its dissolution in electrolyte; and 3) ensuring sufficient contact between sulfur and adjacent conductive substrates. Regarding the role of additive in the cathode, we will review this section from a metal–organic hybrid to all-organic electrocatalysts.

#### Metal–Organic Hybrid Electrocatalysts

In early stages, the main strategy for limiting the shuttle effect of LiPSs was to introduce heteroatoms, metal oxides, metal sulfides, and metal nitrides that suppressed the shuttle effect to a certain extent (Fang et al., 2020). However, these are mostly inorganic bulk materials and only limited surface atoms can be used as trapping sites for LiPSs. In addition, the surface of inorganic bulks usually exists in a variety of crystal structures and planes (Sun et al., 2016), which make it difficult to accurately identify the interaction mechanisms between inorganic bulk materials and sulfur species. Given that the additive is mainly for the interaction with LiPSs, metal-organic materials with a clear molecular configuration and a similar structure of single atomic catalysts will help to further improve the performance of Li−S batteries and lead to a deeper understanding of reaction mechanisms. Before its application, there are several problems to be resolved to obtain highly efficient metal-organic electrocatalysts. An effective strategy is to graft metal-organic materials on carbon frameworks, employing the synergies of the two components.

**FIGURE 2:**
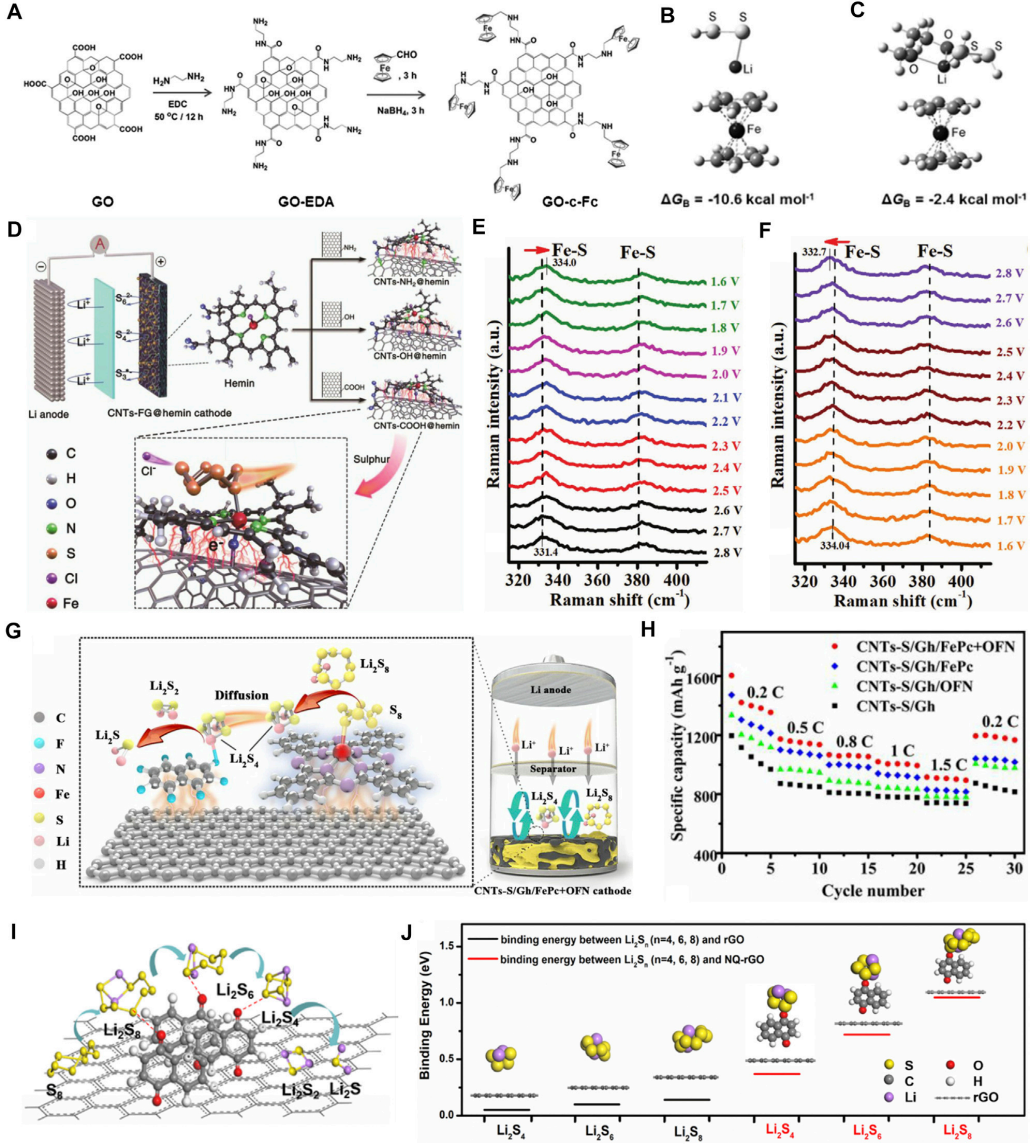
Evolutions of sulfur conversion by organic electrocatalytic additives. **(A)** The schematic synthesis of ferrocene functionalized GO-c-Fc. **(B, C)** Optimized configurations of GO-c-Fc with LiPSs. **(A–C)** Figure reproduced from the data in Mi et al. (2016). **(D)** The Li−S battery based on various CNTs-FG@hemin cathodes (FG = NH_2_, OH, COOH) and the adsorption mechanism of LiPSs on CNTs-COOH@hemin. **(E, F)** *In-situ* Raman spectra of CNTs-COOH@hemin cathode during the discharge and charge processes. **(D–F)** Figure reproduced from the data in Ding et al. (2020). **(G)** The schematic mechanisms of Gh/FePc+OFN for LiPS adsorption/conversion. **(H)** The comparison of rate performances of CNTs-S/Gh, CNTs-S/Gh/OFN, CNTs-S/Gh/FePc, and CNTs-S/Gh/FePc+OFN cathodes. **(G, H)** Figure reproduced from the data in Zhou S. et al. (2020). **(I)** A diagram of NQ-rGO composite and its catalytic effects on LiPSs. **(J)** The optimized configuration and the binding energy of Li_2_S_4_, Li_2_S_6_, and Li_2_S_8_ on rGO and NQ-rGO, respectively. **(I, J)** Figure reproduced from the data in Sun W. et al. (2020).

The metallocene family is composed of central transition metal atoms and organic cyclopentadienyl groups (Goodwin et al., 2021). Among of them, ferrocene, which is aromatic and contains easily replaced sites, shows a high redox activity in the field of electrochemistry (Benecke et al., 2020). Using this advantage, Mi et al. proposed to covalently anchor ferrocene on the graphene oxide, as shown in Figure 2A (Mi et al., 2016). Cation-π interactions would occur between the negatively charged cyclopentadienyl ligand and Li^+^ in LiPSs (Figures 2B,C), achieving effective inhibition of shuttle effect and cycling stability improvement (ultra-low capacity decay rate of 0.014% per cycle) of the Li−S batteries. The relative weak interaction (a few kcal mol^−1^) between the partially positively charged atoms in the molecule and the aromatic groups would lead to limited inhibition of the LiPS migration. Coordinated transition metals in metal phthalocyanine (MPc) or porphyrin (PC), as a contrast, have been recorded by scientists to strongly bind with S in LiPSs through Lewis acidic-basic interactions (Song et al., 2020). MPcs, composed of four isoindole units, are planar conjugated macrocyclic systems that are similar to naturally acquired PCs. Therefore, MPc is also known as tetrabenzazepine porphyrin. This group of materials is characterized by: 1) a special 2D conjugated π electronic structure; 2) good photothermal stability; and 3) abundant molecular structures. In nature, hemin and its derivatives, as the electroactive center of heme protein, are widely used in the fields of oxygen transport and electrocatalysis (Mi et al., 2016). As a pioneer work, our group grafted hemin molecules onto functionalized CNTs and successfully prepared a high-performance biomimetic catalyst for Li−S batteries (Figure 2D) (Ding et al., 2020). We predicted that hemin might have a similar catalytic effect on sulfur, due to the same main group of O and S. Additives by carboxylic CNTs together with hemin can improve the conductivity and dispersion of sulfur by means of π-π conjugated and coordinated bonds. As shown in Figures 2E,F, in order to study the reaction mechanism, *in-situ* Raman characterizations were carried out. The characteristic peaks at 330 and 385 cm^−1^ can be assigned to the vibration of the Fe-S bond. The Fe-S peaks shift to higher wavenumbers during the discharge process and present a reversible tendency upon recharging. These results proved the fact that the use of CNTs-COOH@hemin to form Fe-S bonds with LiPSs promotes the catalytic effect and transformation rate of long-chain LiPSs to S_3_^2-^ (or S_3_^*^), and greatly improves the electrochemical performance. The CNTs-COOH@hemin-based Li−S batteries present an ultra-high initial discharge capacity of 1,637.8 mAh g^−1^ at a current density of 0.2 C, and a good long-term cycling performance (1800 cycles with a mean decay rate of 0.042% per cycle).

Inspired by this efficient catalyst, researchers are conscious of the synergy of multiple active sites on LiPSs. Huang et al. explored a new kind of cobalt phthalocyanine (CoPc) electrocatalytic additives in Li−S batteries (Huang W. et al., 2018). They surveyed the evolution of sulfur species in the reduction process and indicated that long-chain LiPSs could be adsorbed on CoPc. Subsequently, it would receive electrons and gradually be reduced to Li_2_S_2_/Li_2_S precipitation. Furthermore, the interaction mechanism between CoPc and sulfur species was investigated by the XPS measurement. The exposed Co (II) in CoPc can bond with S in LiPSs by Lewis acidic–basic interaction, and the N atom in CoPc can bond with Li, which realizes the chemical binding synergy. These designs promote the redox reaction kinetics of sulfur and improve the long-term cycling stability of Li−S batteries.

All the above works demonstrate that organic electrocatalysts exhibit more obvious catalytic and conversion effects on long-chain LiPSs. From the perspective of the sulfur reduction process, promoting the liquid–solid conversion of soluble short-chain LiPSs to insoluble Li_2_S_2_ and Li_2_S accounts for 3/4 of the total discharge capacity (Li et al., 2019). Therefore, it is the critical aspect of organic electrocatalysis research. More importantly, it has been pointed out that the conversion of liquid–solid phase is the decisive step in the whole sulfur reduction reactions (Zhang et al., 2019a). Along with this line, our group presented a dual-control component strategy for eliminating this barrier by attaching iron phthalocyanine (FePc) and octafluoronaphthalene (OFN) on graphene (CNTs-S/Gh/FePc+OFN) through ultrasonic treatment (Figure 2G) (Zhou S. et al., 2020). The experiments and DFT calculations give clear evidence that FePc can effectively anchor and shear long-chain LiPSs through the Fe-S bond and promote the liquid–liquid phase transformation of LiPSs. Meanwhile, OFN and short-chain LiPSs can interact through Li bonds to accelerate the liquid–solid transformation and promote the nucleation/growth of Li_2_S. As a result, the CNTs-S/Gh/FePc+OFN cathode exhibits an ultra-high initial discharge capacity of 1,604 mAh g^−1^ at a current density of 0.2 C (Figure 2H). After 1,000 cycles at 1.0 C, the mean capacity decay rate is merely 0.055 % per cycle.

#### All-Organic Electrocatalysts

Compared with metal-organic electrocatalysts, all-organic electrocatalysts have no transition metal atoms in center that is more environmentally friendly. However, the absence of metallic active sites should also affect the binding toward LiPSs. In addition, all-organic electrocatalysts usually exhibit poor conductivity and the tendency of agglomeration. The solution for metal-organic electrocatalysts provides a reference for the development of all-organic electrocatalysts. Naphthoquinone (NQ) is widely spread in nature with multiple biological activities (Shi et al., 2020). Sun’s group introduced organic NQ molecules with redox activity onto rGO sheets for Li−S batteries (Figure 2I) (Sun W. et al., 2020), in which the rGO provides conductive networks for facilitating electron transfer and the NQ is used as a catalytic additive. As depicted in Figure 2J, compared with the pristine rGO, the binding energy between NQ-rGO and Li_2_S_4_, Li_2_S_6_, and Li_2_S_8_ is evidently higher. Subsequent tests suggest that the NQ-rGO can inhibit the LiPSs shuttle through chemical bonding between the carbonyl group and LiPSs. As a consequence, the S/NQ-rGO cathode exhibits a high discharge capacity of 525 mAh g^−1^ at 5 C and good cycle stability (maintains 670 mAh g^−1^ at 1 C for 500 cycles). Based on this enlightenment, the realization of synergistic effects of dual-active sites in all-organic electrocatalysts will be helpful to promote the sulfur reduction reactions. Lai et al. used tris (4-fluorophenyl) phosphine (TFPP) as the interface molecular mediator for Li−S batteries (Lai et al., 2019). Triphenylphosphine has been widely reported as a catalyst for organic synthesis reactions (Wang H.-Y. et al., 2020), so do the derivates. They pointed out that the F and P atoms in TFPP can strongly interact with Li^+^ and S_n_^2−^ in LiPSs, respectively, which not only improves the kinetics of liquid–liquid phase transition, but also promotes the formation of short-chain LiPSs (Li_2_S_x_, x = 1, 2, 3, 4) at the interface. The TFPP-based cathode can hold steady after 1,000 cycles at 5 C with a capacity decay rate of 0.042% per cycle.

In all, the main obstacles of using organic electrocatalysts as additives in cathodes are the poor conductivity, agglomeration of small molecules, its dissolution in electrolyte, limited catalytic sites, and the unclear catalytic mechanisms. Although scientists have realized the catalysis of long-chain and short-chain LiPSs, respectively, the introduction of a two-component complex into the cathode system will inevitably reduce the energy density. Therefore, more efficient metal-organic hybrid electrocatalysts still need to be explored. At the same time, the mechanism of synergistic catalysis on the interface needs to be more clearly understood.

### Organic Electrocatalytic Binders

A binder plays a critical role in maintaining the integrity of electrodes and ensuring intimate contact between the active materials and the current collector (Liu et al., 2018). Functional polymer adhesive is the most commonly used binder in lithium batteries. Traditionally, in the Li−S system, the excellent electrochemically stable polyvinylidene fluoride (PVDF) can provide strong adhesion between the current collector and the carbon/sulfur composites (Lacey et al., 2014). However, the toxic, volatile, and flammable organic solvent N-methyl-2-pyrrolidone (NMP) should be used in conjunction with PVDF (Li J.-T. et al., 2017). As a consequence, a higher drying temperature is required to completely remove the solvent. In addition, PVDF shows weak affinity toward LiPSs in a long run (Guo and Zheng, 2020). Therefore, it is necessary to develop organic binders with stronger LiPSs adsorption capacity at the prerequisite of ensuring its high adhesion.

**FIGURE 3:**
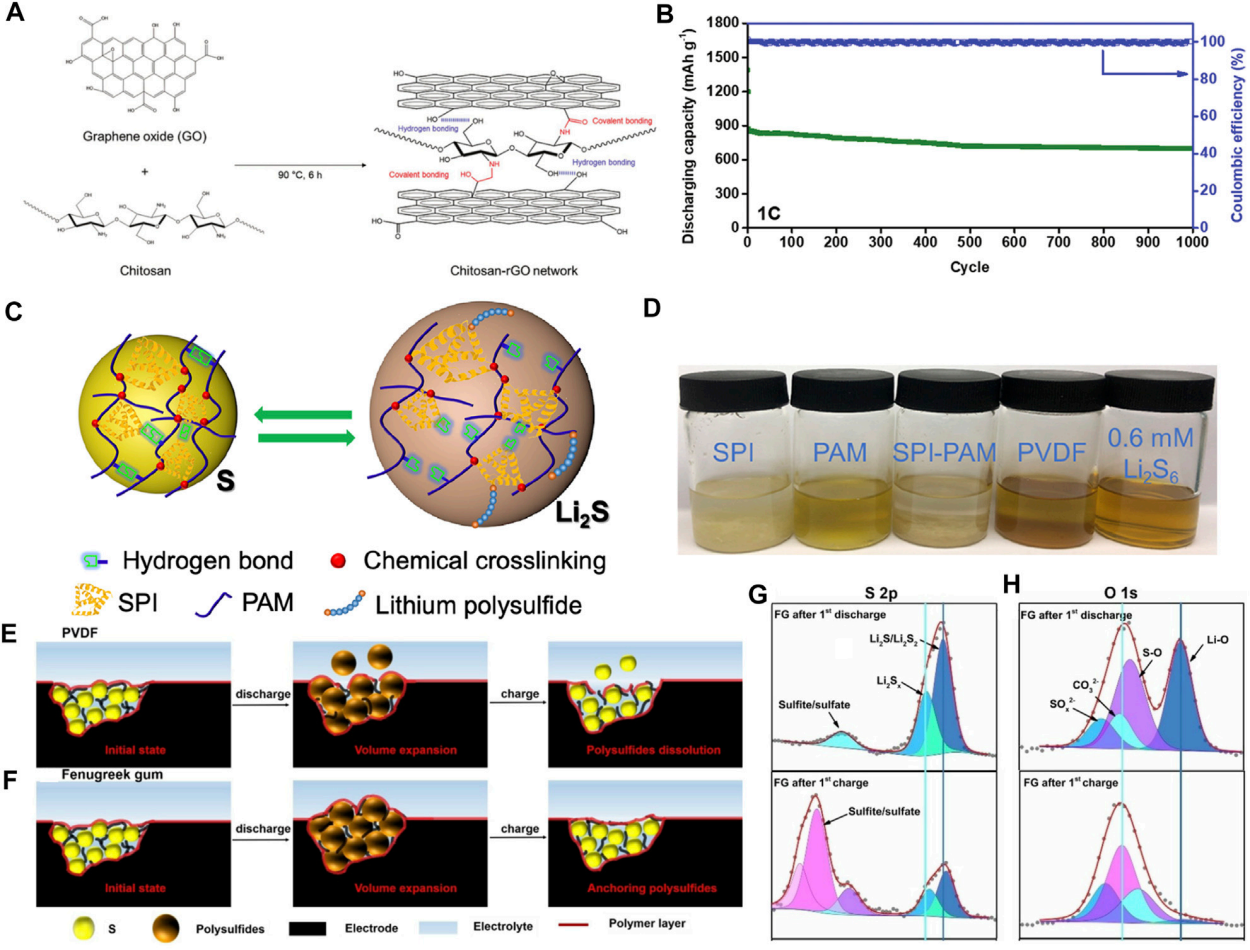
The concepts and mechanisms of organic electrocatalytic binders for Li−S batteries. **(A)** The formation of networked binders by chitosan and rGO composites. **(B)** Long-cycling performance of Chi-rGO-1-based Li−S battery at 1 C. **(A, B)** Figure reproduced from the data in Kim et al. (2020). **(C)** Schematic diagrams of the effects of self-healing SPI-PAM adhesives. **(D)** Visual adsorption experiment of different binders soaked in 0.6 mmol L^−1^ of Li_2_S_6_ solution. **(C, D)** Figure reproduced from the data in Wang H. et al. (2020). **(E, F)** Morphology evolutions of PVDF and FG binders during cycling. **(G, H)** O 1s XPS spectra of FG after initial charge/discharge cycles at 0.1 C. **(E–H)** Figure reproduced from the data in Mo et al. (2020).

Chitosan contains a unique structure of β-(1,4)-2-deoxy-2-amino-d-glucopyranose (Shariatinia, 2019) that can provide fast Li^+^ transport pathways (through abundant hydrogen bonds), high electrochemical stability, flexible matrix, and excellent mechanical properties (Chen et al., 2015). However, its insulating nature causes high internal resistance and low utilization of sulfur in the cathode side, resulting in poor electrochemical performances. In Figure 3A, Kim et al. constructed a new multifunctional binder (Chi-rGO) prepared by combining chitosan and rGO to significantly improve the sulfur conversion reactions (Kim et al., 2020). The new binder, taking the advantage of rGO and electrochemically stable chitosan, offers fast Li^+^ transport channels and functional groups, such as -OH, -NH_2_, -COOH, that exhibit good LiPSs capturing characteristics. These active sites are rationally and uniformly dispersed in the Chi-rGO hosts. Based on their synergistic effect, as shown in Figure 3B, the capacity decay rate of Chi-rGO networked adhesive is 0.016% per cycle for 1,000 cycles at 1 C.

During the charge/discharge process, the sulfur cathode will expand/shrink which may cause collapse of the electrode and the loss of contact between active materials and the current collector, resulting in a rapid decrease in capacity and the shortened lifespan of Li−S batteries. In the field of biology, there is the concept of “self-healing” which is a stable and balanced self-recovery regulation mechanism (Cheng et al., 2019). In Li−S chemistry, it means to immediately repair the defects caused by the volume changes, thus ensuring sufficient electrical contact of active materials and the integrity of electrodes. Inspired by this self-healing mechanism Wang et al. (2017) put forward a new type of adhesive through the copolymerization of methacrylate protein isolate and polyacrylamide (SPI-PAM) (Wang H. et al., 2020). The schematic diagram of the self-healing SPI-PAM adhesive in a Li−S battery is shown in Figure 3C. The SPI-PAM networks are formed by introducing acrylamide groups into chain-like PAM molecules where the involved double cross-linking of intermolecular hydrogen bonds and chemical bonds plays a pivotal role. In this compound, apart from good adhesion, the natural SPI with polar functional groups can be used to anchor LiPSs. Moreover, its appropriate electrolyte absorptivity is conducive to constructing ion/electron transport pathways and maintaining the structural integrity of the cathode. Compared with SPI, PAM, and PVDF, the as-prepared SPI-PAM shows much superior performances. Figure 3D is the visual adsorption experiment of SPI-PAM in LiPSs, which exhibits a strong LiPS anchoring capability. The SPI-PAM-based Li−S batteries can sustain for 400 cycles at 6 C with a capacity decay rate of 0.0545% per cycle.

Compared with the above-mentioned artificially synthesized water-based adhesives, natural organic polymers have abundant functional groups and good chemical affinity toward polar LiPSs, thereby they may take effects in inhibiting or even eliminating the migration of LiPSs (Li Q. et al., 2016). In contrast to traditional PVDF, Mo and colleagues discovered a natural new binder of fenugreek glue (FG) (Mo et al., 2020) (Figures 3E,F) to further improve the electrochemical performance of Li−S batteries. To reveal the interactions between the binder and LiPSs, high-resolution S 2p and O 1s XPS tests were performed (Figures 3G,H). As indicated, the intensities of Li_2_S_2_ and Li_2_S signals are significantly enhanced together with a higher binding energy shift of S 2p peak when discharging the FG-based battery. The O 1s confirmed the Li-O (528.0 eV) and S-O (531.9 eV) interactions between the Li and S atoms in LiPSs and the O atoms in the functional groups of FG. These intense interactions may be responsible for significantly improving electrochemical performances. As a result, the initial discharge capacity of the FG-based battery at 2 C is 900 mAh g^−1^ and retaining 45.6% after 1,300 cycles. Moreover, they tested the tensile properties of the binder. The breaking elongation and strength of the FG binder are as high as 32% and 29.9 MPa, respectively, which are higher than 12.7% and 10.7 MPa of controlled binders (GG), indicating effective suppressing volume expansions by FG.

The recent research progresses of organic electrocatalytic binders are summarized. Although some progress has been made, there are still some problems to be solved. The following research should focus on: 1) improving the mechanical properties of the binder; 2) providing fast ion/electron transport channel; and 3) constructing abundant polar functional groups on binders.

The suppression of shuttle effect and the promotion of sulfur conversion have become hot topics in Li−S systems. Here, we summarize the introduction of organic electrocatalysts in the sulfur cathode as host, additive, and binder and discuss their mechanisms behind boosting the electrochemical performances. Parts of related literatures are listed in Table 1. These organic electrocatalysts can chemically bond with LiPSs through active sites, thereby improving the transfer of Li^+^/S_n_^2−^, promoting the uniform deposition of Li_2_S, and accelerating the kinetics of redox reactions. Despite the achievements, organic electrocatalysts still face several challenges. The prime consideration is the lack of a clear interpretation of catalytic mechanisms by organic electrocatalysts that is critical to design new organic electrocatalysts. To accomplish this task, *in-situ* techniques, as well as innovations at basic materials, need to be followed up.

**TABLE 1:** Some related reports on organic electrocatalytic cathodes for Li−S batteries.

Electrocatalysts	Rate (C)	Initial capacity (mAh g^−1^)	Retention (mAh g^−1^)	S Loading (mg cm^−2^)	Ref
Ni-ZIF-8@CC	0.2	1,080	500th, 715@1C	1.5	Yang et al. (2018b)
Cu-TDPAT	0.1	1,000	500th, 745@1C	1.2	Hong et al. (2018)
ZIF-67-S-PPy	-	-	200th, 599@0.1C	-	Geng et al. (2019)
S@Ni_3_(HITP)_2_-CNT	0.1	1,358.6	100th, 848@0.2C	1.5–1.8	Cai et al. (2019)
LPS-MOF-808@S/GEC	0.5	858 ± 51	100th, 685@0.5C	-	Baumann et al. (2020)
Py-COF	0.5	1,145	220th, 265@5.0C	0.8–1.2	Meng et al. (2018)
COF-ETTA-ETTCA	0.1	1,617	528th, 605@0.5C	1.3	Lu et al. (2020)
EB-COF-PS	0.1	1,136	300th, 468@4.0C	1.5	Zeng et al. (2017)
FCTF-S	0.1	1,296	150th, 833@0.5C	1.3	Xu et al. (2017)
NO-CTF-1	0.1	1,250	300th, 737@0.5C	1.0–1.5	Zhang et al. (2021b)
P-CTFs@rGO	0.05	1,375.2	500th, 920@0.5C	∼1.5	Troschke et al. (2020)
PCNC	4.0	550	800th, 470@4.0C	-	Xiao et al. (2019)
Ferrocene	0.2	1,205	550th, 588@1.0C	1.0	Mi et al. (2016)
CNTs-COOH@hemin	0.2	1,637.8	1800th, 205@1.0C	1.2	Ding et al. (2020)
CoPc	0.1	1,412.2	400th, 719@0.2	1.2	Huang et al. (2018a)
CNTs-S/Gh/FePc+OFN	0.2	1,604	1000th, 423@1.0C	∼1.2	Zhou et al. (2020c)
S/NQ-rGO	0.1	1,340	500th, 670@1.0C	1.4	Sun et al. (2020a)
TFPP	0.2	1,492	1000th, 300@5.0C	0.6–0.7	Lai et al. (2019)
Chitosan-rGO	0.5	1,256	100th, 948@0.2C	0.8–1.0	Kim et al. (2020)
SPI-PAM	0.5	677.6	350th, 678@1.0C	1.3	Wang et al. (2020c)

## Organic Electrocatalysts in Interlayers

During a long-term cycling process, LiPSs in Li−S batteries will inevitably dissolve from the cathode into the electrolyte (Zhu et al., 2021). A traditional polypropylene (PP) separator has large pore sizes of ∼500 nm, which are too large to prevent the migration of LiPSs to the Li anode (Zhao et al., 2018). Even partly blocked, the LiPSs will accumulate on the surface of the separator (Chen et al., 2018). Owing to its nonconductive property, the accumulated LiPSs cannot be converted completely, thereby reducing the sulfur utilization rate. It is noted that the accumulated LiPSs will hinder the Li^+^ transmission channels and eventually lead to a decrease in battery performance (Hwang et al., 2016; Fan et al., 2019). In 2012, for the first time, Su and Manthiram proposed the concept of a “sandwich” structure, that is, a self-supporting multiwalled CNT interlayer was inserted between the cathode and the separator in Li−S batteries (Su and Manthiram, 2012b). Owing to its high conductivity and anchoring effect on LiPSs, the specific capacity and cycling stability are remarkably improved (Su and Manthiram, 2012a). Since then, researchers called this structure as “interlayer.” The ideal interlayer can effectively adsorb LiPSs through the dual effects of physical barrier and chemical bonding that can quickly convert them through electrocatalysis to shorten the residence time of LiPSs in the electrolyte (Wang et al., 2016) and reduce the diffusion of LiPSs to the Li anode (Xu Q. et al., 2021). The interlayer configuration can be designed very thin and lightweight that will not significantly sacrifice the energy density (Dong et al., 2018). As a result, the interlayer can not only reduce the resistance of the sulfur cathode, but also intercept LiPS migration (Jeong et al., 2018). With the help of highly conductive interlayer matrixes (Lai et al., 2019), the effects of catalysts can be further exerted, and the performance of batteries will be greatly improved. Compared to other functional materials, the composite that interlayers loaded with organic electrocatalytic materials possess certain active sites and conductive interlayer matrixes that can exert maximum conversion capacities of sulfur species (Huang et al., 2015). The amount and spatial distribution of active sites in organic electrocatalysts can be rationally regulated. Besides, multiple sites tend to coordinate so as to develop the synergistic effect of adsorption and catalytic conversion. Scientific researchers have done a lot of work along this line of thinking (Hong et al., 2018). In this part, we mainly introduce the application of organic electrocatalytic materials in interlayers from the aspects of chemical binding polysulfide anions, shearing S-S bonds of intermediate species, and the deposition of Li_2_S/Li_2_S_2_ species.

### Chemically Binding LiPSs

**FIGURE 4:**
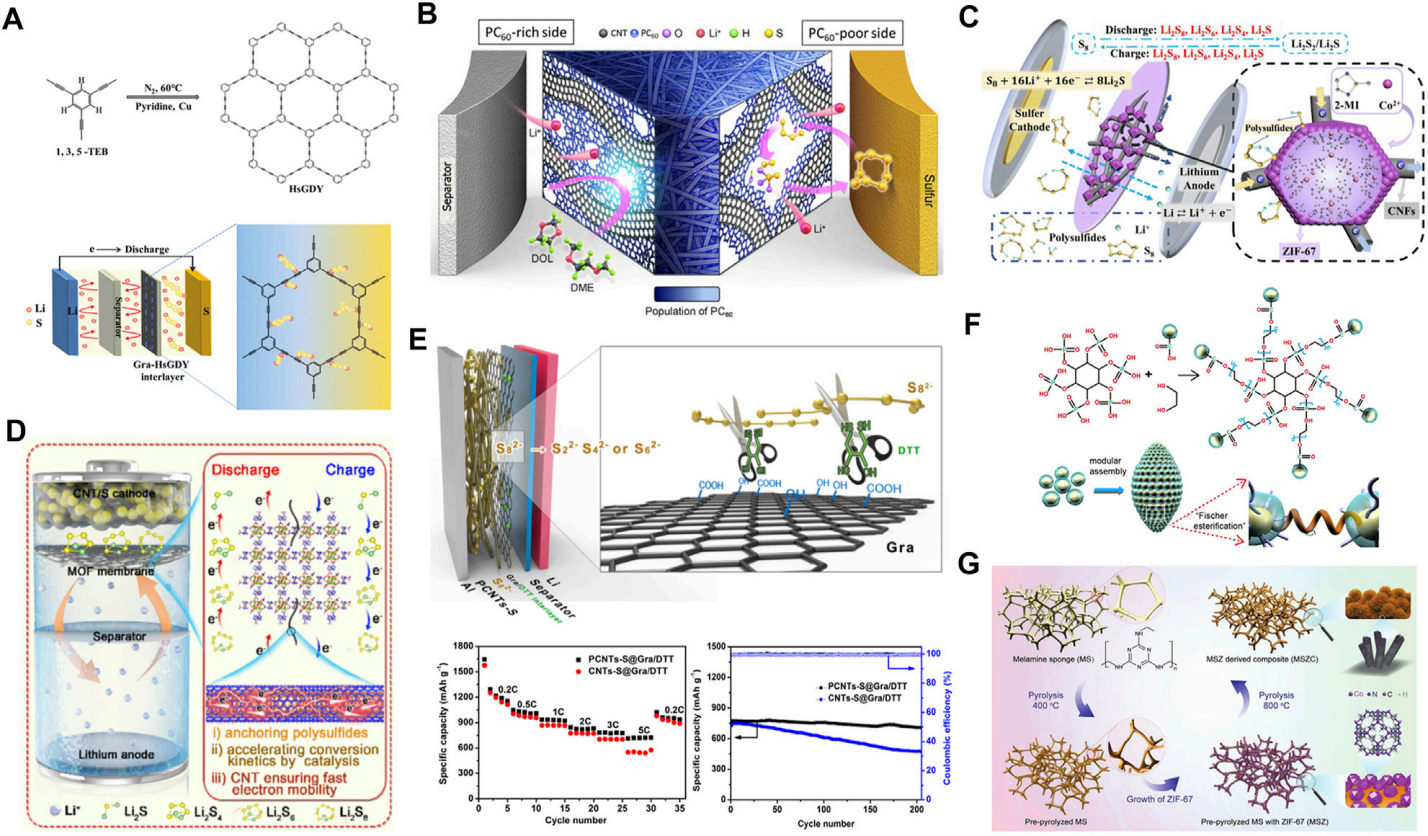
The strategy and performance of organic electrocatalysts for Li−S interlayers. **(A)** Schematic illustration of a Gra-HsGDY interlayer. **(A)** Figure reproduced from the data in Kong et al. (2021). **(B)** The illustration of dual functional and population gradient CNT@PC_60_ interlayer in Li−S batteries. **(B)** Figure reproduced from the data in Hu et al. (2020). **(C)** Mechanism of physical barrier and chemical adsorption for LiPSs by the ZIF/CNFs interlayer. **(C)** Figure reproduced from the data in Li J. et al. (2021). **(D)** The configuration and reaction mechanisms of bimetallic Zr−Fc MOF/CNT interlayer in Li−S batteries. **(D)** Figure reproduced from the data in Wang Y. et al. (2021). **(E)** Schematic configuration of Gra/DTT interlayer and its electrochemical performance. **(E)** Figure reproduced from the data in Hua et al. (2017). **(F)** The modularly assembled XC72 carbon black nanoparticles for the MAXC interlayer. **(F)** Figure reproduced from the data in Ye et al. (2017). **(G)** Typical synthetic process of freestanding MSZC for Li−S interlayers. **(G)** Figure reproduced from the data in Qian et al. (2020).

Recently, our group Kong et al. (2021) designed a sp/sp^2^ hybrid all-carbon interlayer in Li−S batteries (Figure 4A), which consists of graphene (Gra) and hydrogen-substituted graphodiyne (HsGDY) with a specific surface area of up to 2,184 m^2^ g^−1^. The 2D network and the rich pore structure endow HsGDY with a rapid physical adsorption toward LiPSs. Owing to the strong interaction of acetylene bond (C≡C) to Li^+^, C≡C in HsGDY can capture LiPSs and promote the conversion of LiPSs. As a result, the Li−S batteries based on all-carbon interlayer (HsGDY@Gra) exhibit an excellent cycling stability at 1 C for 500 cycles with a decay rate of 0.089% per cycle. In this work, HsGDY is a semiconductor that is adverse to the transfer of electrons and ions. The combination of conductive matrixes and organic electrocatalysts is an effective way to tackle this issue. Tran et al. (2020) induced the generation of organic polymerized fullerene (PC_60_) by the plasma method. Subsequently, the PC_60_ was coated on the CNT matrixes to form a 3D interlayer (CNT@PC_60_). In this work, they adjusted the gradient distribution of organic PC_60_ between the cathode and the interlayer, thus exerting the physical and chemical functions of C_60_-derived free radicals (Figure 4B). According to the CV profiles of the symmetrical batteries, the introduction of CNT@PC_60_ enhances the amplitude of the cathodic and anodic redox peaks, indicating a substantially mitigated polarization. Besides, the Li^+^ diffusion coefficient of CNT@PC_60_, according to CV curves at various scan rates, is significantly improved, suggesting a catalytic effect of PC_60_. Post-mortem analysis of the CNT@PC_60_ interlayer after several cycles is provided by XPS and Fourier transform infrared (FTIR) spectroscopy. As indicated, thiosulfate and polythionate intermediates are clearly detected during the reaction. In addition, insoluble S_2_O_3_^2−^ and CF_3_ species are also identified which can be used as mediators for the transformation of LiPSs. After adding PC_60_, the ratio of sp^2^ carbonyl (C=C) to ether (C-O-C) groups will change significantly, further demonstrating the catalytic effect of PC_60_ on LiPSs. These results prove that the CNT@PC_60_ interlayer can not only physically shield LiPSs, but also act as catalytic fixatives to improve the kinetics of sulfur conversion. The synergistic effect of the CNT@PC_60_ interlayer enables Li−S batteries to maintain an ultra-low attenuation rate of 0.066% at 5 C with a high specific capacity of 829 mAh g^−1^ after 400 cycles (Hu et al., 2020).

As narrated in the sulfur cathode section, MOFs also show prospects in the interlayers. Li J. et al. (2021) proposed a Co-based MOF (ZIF-67) interlayer by its *in-situ* growth on carbon nanofibers (CNFs) (Figure 4C). The 3D CNFs provide interconnected conductive frameworks between ZIF micro-reactors, constructing channels for electron/Li^+^ transfer. The characteristic XPS peaks of Co^2+^ and S_n_^2-^ with and without cycling show remarkable shifts, indicating the interaction between Co^2+^ and LiPSs. Hence, the shuttle effect of LiPSs was effectively restrained, leading to the rate and stability improvement of batteries (He Y. et al., 2018). Furthermore, the functionality of MOFs can be modified through adjusting central atoms. By using a simple wet chemical method, Tian’s group directly synthesized a thin-layer MOF nanosheet {Cu_2_(CuTCPP) [TCPP = 5, 10, 15, 20-tetrakis (4-carboxyphenyl)porphyrin]} without any surfactants. Assembling these ultra-thin nanosheets via a simple vacuum filtration method, they obtained a highly oriented flexible membrane with good mechanical properties. The as-synthesized Cu_2_(CuTCPP) exhibits abundant microporous for blocking LiPSs and high conductivity. Furthermore, the XPS and FTIR measurements demonstrate the strong interactions between Cu_2_(CuTCPP) and LiPSs that is conducive to capture LiPSs (Tian et al., 2019). To further increase the active sites, bimetallic-centered MOFs have been studied intensively. Wang R. et al. (2021) fabricated a bimetallic Zr-Fc MOF-based nanosheet as a multifunctional interlayer by combining 2D ferrocene (Fc) MOF and CNTs via a simple vacuum filtration method (Figure 4D). Zr-Fc MOF can interact with the negatively charged LiPSs through the positively charged open metal sites of Zr-Fc MOF. As a result, the Zr-Fc MOF can inhibit LiPSs by electrostatic attractions and chemical anchorings. Specifically, the Zr^2+^ and Fe^3+^ in Zr-Fc MOF play a good electrocatalytic effect on the redox kinetics of LiPSs. The entangled CNTs throughout the Zr-Fc MOF nanosheets promote electronic conductivity and the capture of Zr-Fc MOF. Owing to the capture–catalysis–conversion effect, the Zr-Fc MOF/CNT interlayer exhibits a significantly enhanced rate and cycling performance.

Nevertheless, the role of different chemical groups in reacting with LiPSs and the effects of bottom-up assembly of MOFs in intercalated membranes on the diffusion of LiPSs have yet to be fully understood. Therefore, it is still a challenge for optimizing the interlayer through the design of functional sites and microstructures (Song et al., 2017). Guo’s group proposed an orderly multilayered MOF (UiO-66) for the Li−S interlayer (Guo S. et al., 2021). In this case, the diffusion of LiPSs will be restricted in the ordered channels that are formed by the MOF and Gra sheets. The interaction between LiPS molecules with different functional groups was studied. Owing to the presence of functional groups (-OH, -COOH, and -NH_2_), the as-fabricated MOFs are endowed with high adsorption capabilities toward LiPSs that can be ascribed to the Lewis acidic–basic interactions. The XPS spectra present the opposite shift of the S_B_^2−^ peak in LiPSs and the N 1s peak in UiO-66-NH_2_, indicating the interaction of the LiPSs and -NH_2_ groups. Therefore, abundant amino groups in UiO-66-NH_2_ should promote the adsorption of LiPSs, which explains its highest adsorption capacity. The electrochemical tests demonstrate the superior performance of the ordered interlayer than the disordered one. As a consequence, the discharge capacity and cycling stability by using UiO-66-NH_2_ interlayers are significantly improved.

### Shearing the S−S Bond

Compared with traditional catalysts, biocatalysis or biomimetic catalysis is more efficient under mild conditions. Biological reagents such as vitamin C (VC), glutathione (GSH), and dithiothreitol (DTT) can quickly cleave S−S bonds at room temperature. For the DTT and GSH reductants, the active protons are lost under weakly alkaline conditions, and the exposed thiolate anion becomes active which would react with the oxidative S−S bond to break it. For the VC, the active site locates at the position of 2, 3-enediol. Under weakly alkaline conditions, it would lose the active protons. The exposed oxygen anions become active and can reduce the S−S bond, thereby breaking it. In Li−S systems, the biocatalysis or biomimetic catalysis may also play effective roles in solving the slow reaction kinetics of sulfur conversion. In 2017, our group Hua et al. (2017) first proposed the concept of shearing sulfur by biomimetic dithiothreitol (DDT) catalysts. The catalyst is composed of a porous CNTs/S cathode (PCNTs-S) and a lightweight Gra/DTT interlayer (Figure 4E). The concept involves DTT-assisted cutting of S−S bond in LiPSs. The CV tests indicate that the introduction of the Gra/DTT interlayer can reduce the voltage polarization and enhance the reversibility of batteries. In addition, EIS measurements suggest the lowest impedance of Gra/DTT interlayer during the repeated cycling. As a result, the battery with Gra/DTT interlayer endows an initial discharge capacity of 1,643 mAh g^−1^ at 0.2 C, and remains 880 mAh g^−1^ at 1 C for 400 cycles with a capacity decay rate of 0.029% per cycle.

### Accelerating the Precipitation of Li_2_S_2_/Li_2_S

Pristine S_8_ and the final discharged products of Li_2_S_2_/Li_2_S are electronically and ionically insulated, resulting in slow electrochemical reactions, especially at high current densities. Therefore, it is urgent to explore how to accelerate the deposition of Li_2_S_2_/Li_2_S. Ye’ group proposed a modularly assembled interlayer by condensing Vulcan XC72 carbon black (XC) monomer into an ellipsoidal microstructure to assemble XC72 carbon black nanoparticles (MAXC) (Figure 4F). In a high-resolution transmission electron microscope (HR-TEM), the MAXCs are closely connected to each other. This cross-linked structure can provide 3D channels to promote the transfer of electrons and ions. The arrangement of XC nanoparticles in MAXC can promote the adsorption of LiPSs and its redox reactions which can also act as a new current collector, thereby improving the reutilization of the absorbed LiPSs. Besides, micropores in MAXC interlayers can uniformly distribute the flux of Li^+^ to suppress the uneven growth of Li dendrite and ultimately promote the uniform deposition of Li_2_S at the cathode side (Ye et al., 2017). After that, the same team modified the Co-based MOF and synthesized a 3D functional interlayer (Qian et al., 2020) of MS-ZIF-67 (MSZC) by pyrolyzing the ZIF-67-loaded melamine sponge (MS) (Figure 4G). The unique geometric structure of MSZC is helpful for the penetration of electrolyte. Co nanoparticles in MSZC are regarded as catalysts to promote the conversion from LiPSs to Li_2_S_2_/Li_2_S. A similar strategy was reported by Li et al. that another Co-based MOF (C-ACF) was employed. In this configuration, Mo_2_C decorated N and S co-doped carbon framework (N, S-Mo_2_C) is regarded as the host of interlayer. By combining the C-ACF and N, S-Mo_2_C, a composite interlayer (N,S-Mo_2_C/C-ACF) was prepared for Li−S batteries (Li H. et al., 2020). This N,S-Mo_2_C/C-ACF interlayer shows an ultra-fast wetting ability in the electrolyte and a large Li^+^ transfer number. Besides, the formation of a Li-X (X = N, S) bond suggests the chemically anchoring effect of the N and S atoms toward LiPSs (Zhang et al., 2020b). Mo atoms with abundant empty orbitals can provide favorable conditions for LiPS redox reactions. As a result, the batteries with the N, S-Mo_2_C/C-ACF interlayer can deliver excellent rate performance in a wide temperature range. At a high rate of 5 C, the specific discharge capacity of 405, 630, and 670 mAh g^−1^ was obtained at 5, 30, and 55 C, respectively. A stable long-term cycling performance at 1 C for over 600 cycles was acquired with a low capacity attenuation of 0.08% per cycle.

## Organic Electrocatalysts for Separator Modifying

Separator, the indispensible component of a Li−S battery, plays a pivotal role in isolating the anode and the cathode, so as to prevent short circuits (Jin et al., 2021). Generally, the inactive separator does not directly participate in the electrochemical reactions, but it works in processes such as ion/mass transport, which is closely related to the internal resistance, rate performance, and the cycling performance (Li M. et al., 2017). In this system, traditional PP separators cannot fully realize the advantages of Li−S batteries, causing decreased discharge capacity and Coulombic efficiency (Mathew et al., 2020). Similar to the regulation of interlayers, traditional separators can be reasonably functionalized and modified through controlling the pore structure, ion conductivity, adsorption, and catalysis of LiPSs (Qi et al., 2020). In recent few years, reports on the subject of separator modifications by organic electrocatalysts have proliferated. Herein, we will introduce the effects by separator decorations in this field from the following aspects: chemical bonding and catalytic conversion of soluble LiPSs, and guiding the deposition of insoluble Li_2_S_2_/Li_2_S.

### Chemically Binding LiPSs

**FIGURE 5:**
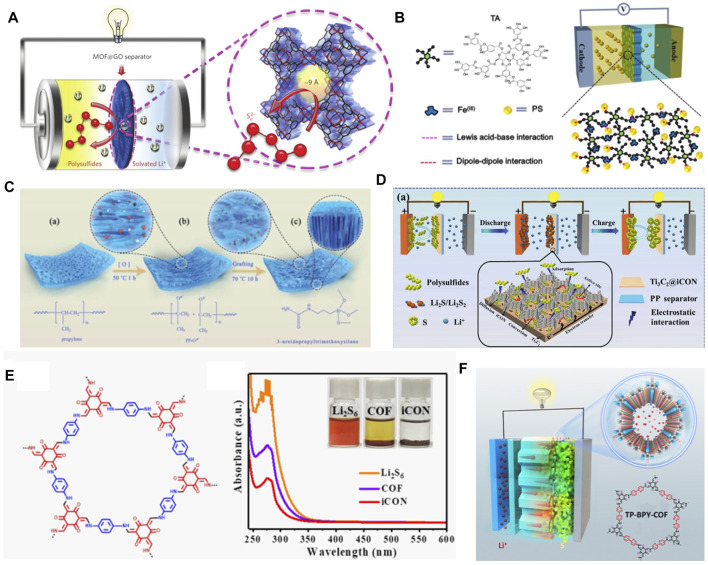
Typical strategies for separator modifying by organic electrocatalysts in Li−S batteries. **(A)** The concept of ionic sieve by MOF@GO modified separator for inhibiting the dissolution of LiPSs. **(A)** Figure reproduced from the data in Bai et al. (2016). **(B)** Schematic illustration of TA/Fe^3+^-PP separators. **(B)** Figure reproduced from the data in Zhang H. et al. (2018). **(C)** The preparation process of the PP-O^x-^-U functionalized separators. **(C)** Figure reproduced from the data in Zhou H. et al. (2020). **(D)** Effects of Ti_3_C_2_ MXenes in Ti_3_C_2_@iCON modified separators for improving the electrochemical performance. **(E)** The structure of charge-neutral COF containing β-ketoene units and UV-vis absorption spectra and digital photographs (inset) of Li_2_S_6_ solution after the addition of iCON. **(D, E)** Figure reproduced from the data in Li P. et al. (2021). **(F)** The design strategy of TP-BPY-COF-modified separators. **(F)** Figure reproduced from the data in Xu et al. (2019).

A feasible solution for the dissolution of LiPSs is to block its shuttle path. The concept “ion sieve” for separating target ions from the solution is regarded as the ultimate objective. MOFs with large surface areas (Hong et al., 2019), highly ordered structure, and adjustable pore size are considered to be suitable ion sieves for mitigating the shuttle of LiPSs. The insulation of MOFs is satisfied with the separation of electrons between the cathode and the anode. Because of this point, the accumulated LiPSs on separators cannot be effectively converted. Therefore, functional additives should be grafted on these MOFs to facilitate their conversion. Bai et al. proposed to design an organic–inorganic hybrid composite on separators by a MOF of Cu_3_(BTC)_2_ (HKUST-1) and graphene oxide (GO) (denoted as HKUST-1@GO) (Bai et al., 2016) (Figure 5A). In this system, Cu cations in HKUST-1 and polysulfide anions can chemically interact. The window size of HKUST-1 is comparable to that of soluble Li_2_S_n_ (4 <n ≤ 8) that may provide physical barrier for the diffusion of LiPSs. The synergy of these physical and chemical interactions is contributed to a high performance Li−S battery. The strategy, combining the advantages of MOFs and conductive polymers, has shown great prospects in the field of separator modifications. Here, the polymers, such as polydimethylsiloxane (PDMS), polyethylene glycol (polyethylene glycol), or cellulose with polar functional groups (Si-O, C-O-C, or -OH) (Suriyakumar et al., 2018), were proved to inhibit the shuttle effect of LiPSs. Gao et al. designed a three-layer separator (Gao et al., 2021) with stepped channels by the preparation of MOFs (Zr-, Cu-, Zn-, and Ce-based MOFs) into various organic polymers. In comparison, they confirmed the smallest contact/electrochemical impedance of the three-layer structured separators, which shows faster electronic transfers at the electrode/electrolyte interface. In addition, the Li^+^ migration number of this modified separator, calculated by chronoamperometric curves, is the largest one. These unique characteristics contribute to suppressing the polarizations through a reversible and rapid Li^+^/electrolyte coupling transfer, leading to the battery performance improvements (Wang et al., 2015).

To further improve the conductivity of the separators, the other set of experiments was conducted by Zhang H. et al. (2018); they coated a tannic acid (TA)/Fe^3+^ composite on traditional PP separators (TA/Fe^3+^-PP) (Figure 5B). TA, a kind of plant polyphenol that contains a central glucose core and a digalloyl ester shell, can provide multi-dentate ligands to coordinate Fe^3+^ and spontaneously form polyphenol metal complexes. The design of the TA/Fe^3+^ compound is based on the dipole–dipole interactions of abundant oxygen-containing groups in TA and Lewis acidic–basic interactions of Fe^3+^ toward LiPSs. The presence of oxygen-containing groups endows the modified membrane with an excellent hydrophilicity for the electrolyte infiltration. These strategies tend to reduce the transfer resistance of Li^+^ and enhance the whole conductivity, thus improving the battery performance, especially at high current rates.

If the modifier of separators does not cause significant increases in weight and volume, it will not make too much impact on the overall energy density. For this consideration, He’s group fabricated a well-arranged hollow Co_9_S_8_ array on a Celgard separator (Co_9_S_8_-Celgard) (He J. et al., 2018) as an efficient barrier layer for LiPSs. The Co_9_S_8_ array is tight and light-weight which can retain the advantages of high-volume/mass energy density of Li−S batteries. The low-voltage hysteresis and highly reversible CV profiles suggest the robust configuration of Co_9_S_8_-Celgard and the reversible sulfur redox reactions. The Co=S bond was detected by FTIR after several cycles that clarified the interacting model between Co_9_S_8_-Celgard and LiPSs. Co 2p_3/2_ peak shifts are consistent with that of Co_9_S_8_-Celgard-Li_2_S_6_ solution from the *ex-situ* disassembled batteries. The Co_9_S_8_-Celgard shows strong affinities toward LiPSs due to the chemical adsorption of Co_9_S_8_ to LiPSs. Apart from this strategy, a polymerization method for separator modification was first proposed by Zhou H. et al. (2020). In their design concept, the PP membrane was grafted with 3-ureapropyl-trimethoxysilane (PP-O^x-^-U) by the single-side chemical tailoring method (Figure 5C), which contains abundant active sites. In XPS tests, they found that the peaks of N 1s and Si 2p in PP-O^x-^-U shift oppositely to the S 2p peak, indicating the synergistic effect of amide and siloxy group on the PP-O^x-^-U separator.

### Catalytic Conversion of LiPSs

High conductive and N-rich carbon nitrides (c-CN) have aroused extensive research in Li−S batteries. Based on a magnetic MOF (Ni(HNCN)_2_), Cai et al. (2020) constructed a bamboo-like c-CN modifier with a sheet resistance of 3.1 Ω sq^−1^ and a N content of ≥10.8 at%. According to DFT simulations, this N-doped c-CN shows high binding energy with LiPSs due to the formation of the Li−N and Li−S bonds. Furthermore, they demonstrated that Ni atoms in Ni(HNCN)_2_ can catalyze the conversion of LiPSs. As a result, the separator can effectively alleviate the shuttle of LiPSs and promote the conversion of sulfur species. The Li−S batteries by using c-CN show good rate capability (1,145.7 and 996.8 mAh g^−1^ at 1.0 and 2.0 C, respectively) and cycle performance (a mean decay rate of 0.088% at 2.0 C for 400 cycles). The other typical catalysts for the LiPS conversion are MXenes that contain a family of 2D transition metal carbides/nitrides. MXenes, marked by their high conductivity and amphiphilic toward LiPSs, have attracted large amounts of attentions in Li−S batteries. However, MXenes suffer from severe agglomerations due to the van der Waals forces and hydrogen bonds, resulting in the loss of specific surface area for LiPSs. Li et al., for the first time, reported a modified separator by combining MXenes (Ti_3_C_2_) with COFs (Ti_3_C_2_@iCON-PP) (Li P. et al., 2021). Specifically, as shown in Figure 5D, the Ti_3_C_2_ nanosheets were uniformly loaded on the integration of guanidinium-based ionic-covalent organic nanosheets (iCON), forming Ti_3_C_2_@iCON. The CV tests suggest that the addition of Ti_3_C_2_@iCON to the PP separator enhances the reversibility and kinetics of electrochemical reactions. When the Ti_3_C_2_@iCON is soaked in the Li_2_S_6_ solution (Figure 5E), the characteristic peaks of the terminal and bridge sulfur species show noticeable linkage shifts with Ti 2p peaks, indicating the transfer of electrons from LiPSs to Ti_3_C_2_. They noted that the good catalysis of Ti_3_C_2_ can effectively accelerate the conversion of LiPSs.

### Promoting the Precipitation of Li_2_S_2_/Li_2_S

As put forward, the reduction of soluble Li_2_S_4_ to Li_2_S deposition accounts for three-fourths of the total capacity. The liquid–solid transition is seriously hindered in most Li−S batteries. Here, a redox active COF of TP-BPY-COF (1,3,5-triformylphloro-glucinol-1,3,5-triformyl-phloro glucinol-covalent)-based separator, proposed by Xu et al. (2021a), was used to take effects in this stage (Xu et al., 2019), as shown in Figure 5F. The TP-BPY-COF plays multiple roles where the 1D pore provides a rapid transport route for Li^+^. The involved phenolic hydroxyl groups work as Lewis acidic to improve the chemical absorption of LiPSs. Furthermore, pyridine nitrogen in TP-BPY-COF frameworks can interact with Li^+^ through a dipole–dipole interaction, forming Li bonds, which is beneficial to the formation of final Li_2_S_2_/Li_2_S products. As a consequence, the batteries with TP-BPY-COF exhibit good cycling stability (826 mAh g^−1^ at 1 C after 250 cycles) and excellent Coulombic efficiency (close to 100%).

Apart from the three aspects for separator modifications, more problems can be solved by organic electrocatalysts. As far as a Li−S battery concerns, the low flash point and poor mechanical strength of traditional PP separators can hardly survive under extreme environments, bringing about safety hazards. Based on the poly (metaphenylene isophthalamide) (PMIA) membrane and the *in-situ* formed cobalt-containing zeolite imidazole (ZIF-L) skeleton, Zhang T. et al. (2021) devised a heat-resistant and nontoxic functional graded separator (Z-PMIA). Compared with PP separators, the presence of Co 2p_1/2_ and Co 2p_3/2_ in Z-PMIA can promote the reversible reduction of LiPSs (Wang and Li, 2021). When heating, the PP membrane shows obvious thermal shrinkage in 4 s, while the PMIA membrane exhibits a smaller shrinkage even after 60 s, which can be attributed to the higher decomposition temperature of PMIA (∼400°C). They indicated that the Li−S batteries with Z-PMIA separators exhibit a lower charge transfer resistance and faster reaction kinetics. Owing to the uniform pore distributions and high electrolyte absorptions, the PMIA separator obtains a high ion conductivity that is conducive to the high flux transmission of Li^+^. The initial discharge capacity of the Z-PMIA-based batteries is as high as 1,391.2 mAh g^−1^ with a slow capacity decay of 0.033% per cycle. Increasing the sulfur loading to 9.23 mg cm^−2^ and reducing the electrolyte/sulfur ratio (E/S) to 8 ml/g, the battery can still achieve high electrochemcial performances at a high working temperature of 80°C. These works highlight the organic electrocatalysts in the commercialization of Li−S batteries.

## Organic Electrocatalysts as Redox Mediators in Electrolyte

Traditionally, a large number of nonactive additives in Li−S batteries severely reduce their energy density as a whole. When redox mediators (RMs) with appropriate potential and Fermi energy level are introduced into the electrolyte, they can transfer interfacial charges on the surface of electrode, promoting the redox reactions. In this section, we will review the functionalities of redox mediators in electrolyte for improving the performance of Li−S batteries.

### Reduction Process

**FIGURE 6:**
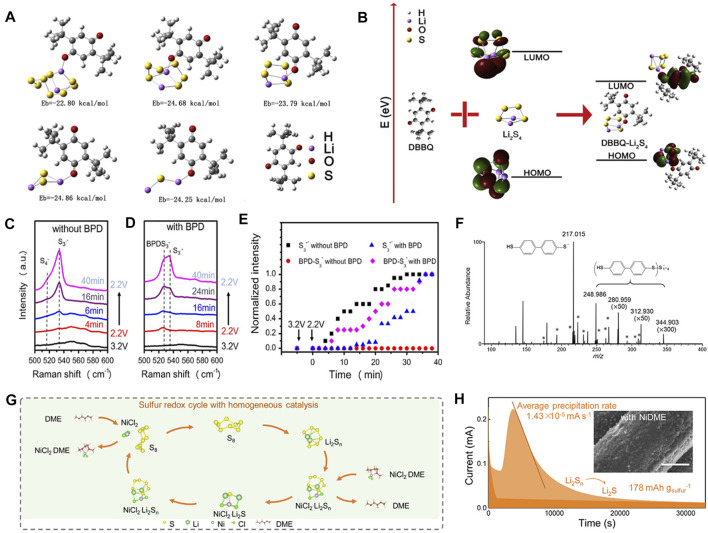
Redox mediators by organic electrocatalysts in electrolyte for the reduction process of Li−S batteries. **(A)** The stable configurations of DBBQ and DBBQ-Li_2_S_x_ (x = 1, 2, 4, 6, 8) with various binding energies. **(B)** The LUMO and HOMO energy levels of electrolyte before and after the reactions between DBBQ and Li_2_S_4_. **(A, B)** Figure reproduced from the data in Wang Z. et al. (2020). **(C, D)** *In-situ* Raman spectra of S/C cathodes with/without BPD additives. **(E)** The peak intensity of S_3_^•^ and BPD-S_3_^−^ as a function of time under the presence/absence of BPD additives. **(F)** (**−**)ESI mass spectrum of the same 5 mM “Li_2_S_4_” solution with 5 mM BPD. **(C–F)** Figure reproduced from the data in Wu et al., 2017. **(G)** Schematic illustration of NiDME additives to catalyze LiPSs in Li−S batteries. **(H)** Potentiostatic discharge curve of Li_2_S deposition under the addition of NiDME. **(G, H)** Figure reproduced from the data in Luo et al. (2020).

Owing to the slow charge transfer rate and conversion kinetics, soluble LiPSs would accumulate in an electrolyte, resulting in the block of reaction path and significant reduction of electrochemical performance (Yan et al., 2016). The conversion of LiPSs is a “short board” in the sulfur redox process. It is expected that the rapid reduction of sulfur can be realized by using redox mediators, as proved by great numbers of reports (Gupta et al., 2020). The frontier molecular orbital (FMOS) energy level of a compound largely determines its redox ability (Kwon et al., 2018) that can be adjusted by introducing redox mediators into electrolyte. It has been reported that 2, 5-di-tert-butyl-1,4-benzoquinone (DBBQ) can form a Li−O bond with LiPSs to regulate the molecular orbital. Wang’ group obtained the binding energy and optimized structure model between DBBQ and LiPSs through DFT calculations (Figure 6A) (Wang Z. et al., 2020). Under the guidance, by the use of DBBQ additives, LiPSs can be captured and covalently fixed. The higher HOMO and lower LUMO energy levels of electrolyte endow the complex with high redox properties (Figure 6B). As a result, the battery with a DBBQ additive can sustain for 100 cycles at a sulfur area density of 7 mg cm^−2^. As the other case, dimethyl disulfide (DMDs) that contains the S−S bond is expected to provide additional theoretical capacity in Li−S batteries by breaking the S−S bond (Chen S. et al., 2017). Chen et al. found that the DMDs can react with sulfur to form soluble methyl terminated LiPSs intermediates and further be reduced to organic lithium sulfide in the discharge process (Chen et al., 2016).

Despite the improvement of actual energy density by these approaches, several issues are still unsolved, for example the corrosion of Li anode by LiPSs (Zhang L. et al., 2018). To handle this issue, Wu et al. introduced biphenyl-4,4′-dithiol (BPD) into the electrolyte (Wu et al., 2017). The *in-situ* Raman spectra suggest the formation of BPD-LiPSs complex during the discharge stage, indicating the mediator role of BPD (Figures 6C–E). Furthermore, it has also been confirmed in the mass spectrometry that the peaks at m/z = 217.015, 248.986, 280.959, 312.930, and 344.903 can be attributed to BPD, (BPD)S^−^, (BPD)S_2_^−^, (BPD)S_3_^−^, and (BPD)S_4_^−^, respectively, indicating that short-chain LiPSs can react with BPD to form stable complexes (Figure 6F). These complexes can inhibit the formation of S_3_^−^ or S_4_^−^ in the electrolyte and improve the utilization of sulfur. In addition, they pointed out that BPD can also promote the formation of a stable SEI layer on the Li anode and suppress Li dendrites.

Large amounts of literatures have repeatedly demonstrated that the liquid–solid phase conversion of soluble Li_2_S_4_ to insoluble Li_2_S_2_/Li_2_S is the speed-determining step of the entire sulfur reduction process. Therefore, it is important to control the deposition of Li_2_S (Yang X. et al., 2019). Based on the above analysis, Luo et al. found that the addition of nickel glycol dimethyl ether (NiDME) into the electrolyte can reduce the activation energy (Ea) of the sulfur redox reactions, thus improving the electrochemical performance (Figure 6G) (Luo et al., 2020). The sulfur species with the NiDME additive can deliver faster deposition rate and higher capacity (178 mAh g^−1^) (Figure 6H). Besides, the Li_2_S can be deposited evenly on the surface of carbon fibers without noticeable agglomerations. They concluded that the chemical interactions between the NiDME additive and LiPSs accelerate the redox reaction kinetics, regulate the nucleation and deposition of Li_2_S, and efficiently improve the utilization of sulfur.

### Oxidation Process

**FIGURE 7:**
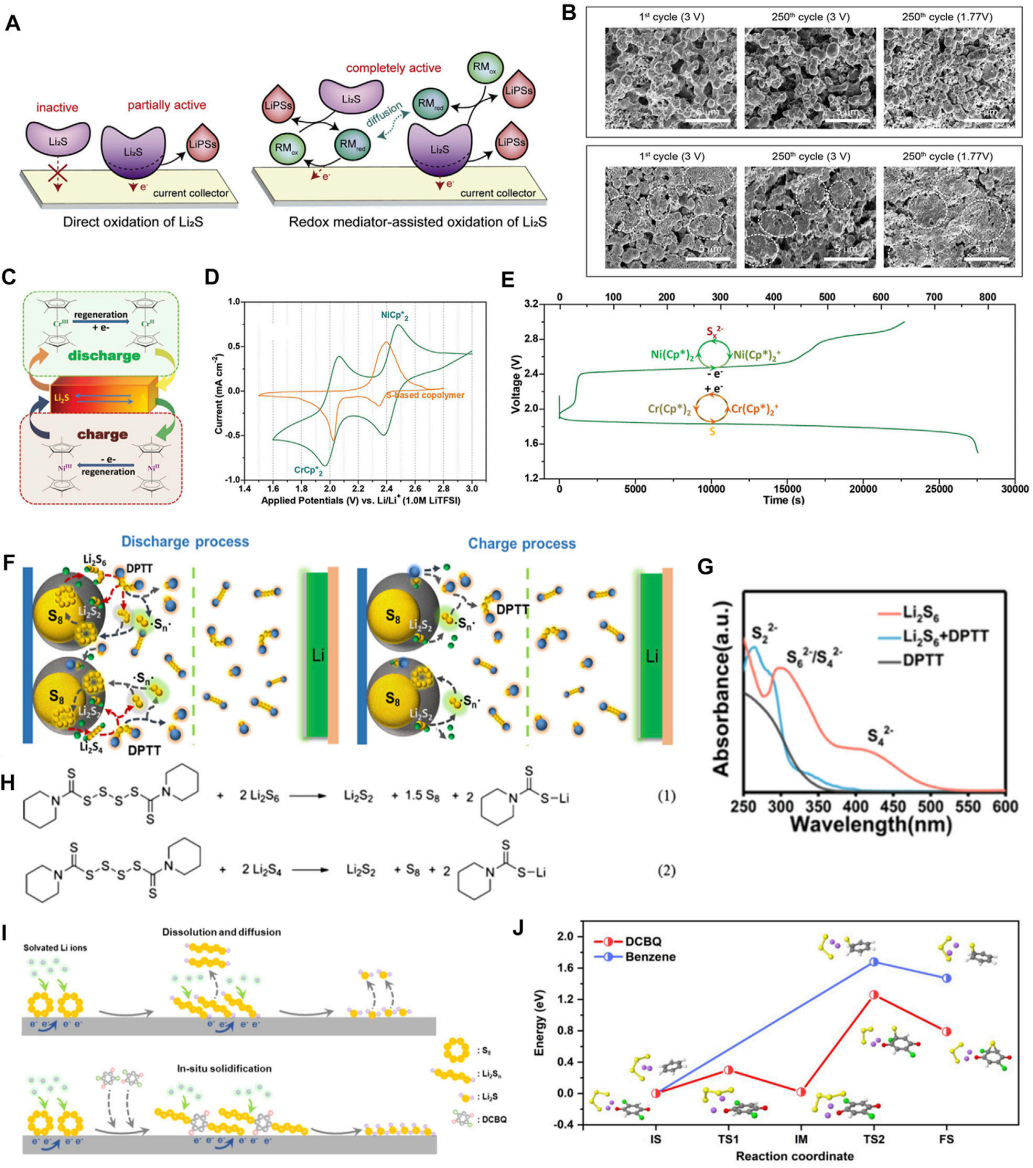
The oxidation processes and specific solid–solid transformations of LiPSs by organic electrocatalytic redox mediators in electrolyte. **(A)** The comparison of direct oxidation and redox mediator-assisted oxidation of Li_2_S in Li−S batteries. **(B)** Morphology evolutions of Li_2_S precipitation on the surface of cathode with and without AQT additives. **(A, B)** Figure reproduced from the data in Tsao et al. (2019). **(C, D)** The schematic illustration of the oxidation–reduction process of Li_2_S catalyzed by CrCp^*^_2_ and NiCp^*^_2_ and their corresponding CV profiles. **(E)** The galvanostatic charge/discharge curves of a Li−S battery under the presence of CrCp^*^_2_ and NiCp^*^_2_. **(C–E)** Figure reproduced from the data in Li et al. (2015). **(F–H)** The proposed chemical reaction paths between DPTT and LiPSs. **(G)** The effect of DPTT mediator by UV-vis absorption spectra. **(F–H)** Figure reproduced from the data in Xiang et al. (2019). **(I)** The diagrams of sulfur reduction processes with/without DCBQ additives. **(J)** Potential energy levels of Li_2_S_4_ reduction process under the presence/absence of DCBQ additive. **(I, J)** Figure reproduced from the data in Chen et al. (2021).

As a reverse reaction process, accelerating the solid–liquid–solid conversion of insoluble Li_2_S to soluble LiPSs and finally to S_8_ is also critical to obtain highly reversible Li−S batteries (Nazar et al., 2014). As shown in Figure 7A, Tsao’s group took the anthraquinone (AQT) linked polyether chain as a redox mediator, which exhibited suitable redox potential, high stability, and promoted the oxidation capability of Li_2_S (Tsao et al., 2019). Figure 7B is the SEM images of the cycled cathode. In the absence of AQT, Li_2_S is unevenly deposited on the electrode after 250 cycles that hinders the charge transfer at the electrode/electrolyte interfaces, resulting in sluggish reaction kinetics. In sharp contrast, the morphology of Li_2_S films is almost unchanged after the introduction of AQT. Taking this advantage, the initial discharge capacity at 0.5 C can reach up to 1,300 mAh g^−1^.

Nevertheless, it is expected to further improve the reaction kinetics and performances of the Li−S battery by simultaneously synergistic catalysis of the reduction and oxidation processes. As a typical case, Chromocene (CrCp^*^_2_) and Nickelcene (NiCp^*^_2_) as redox mediators were added in the electrolyte, as shown in Figure 7C (Li et al., 2015). The reversibility of redox reactions was evaluated by CV experiments (Figure 7D) where the reduction peak of CrCp^*^_2_ is ∼1.96 V and the oxidation peak of NiCp^*^_2_ is ∼2.49 V which is located in the redox potential window of sulfur. In this process, CrCp^*^_2_ and NiCp^*^_2_ promote the reduction and oxidation, respectively. Figure 7E provides the galvanostatic charge/discharge tests. To further optimize the performance, inspired by high biocatalysis of enzymes under the assistance of coenzyme in natural systems (Krishnan et al., 2020), the redox mediator (EV(ClO_4_)_2_) was used as the coenzyme to react with insoluble S/Li_2_S precipitation to form soluble LiPSs (Ye et al., 2021). As a result, the as-fabricated Li−S battery demonstrated a high sulfur utilization of 61% under the sulfur loading of 5.6 mg cm^−2^ and an E/S of 5 μl mg^−1^.

Despite the effective improvements by the addition of multicomponent mediators, more work needs to illustrate the interactions among these components. Therefore, for simplicity, the development of a single redox mediator for simultaneous catalysis of reduction and oxidation of sulfur has become a research hotspot. Dipentamethylenethiuram tetrasulfide (DPTT), a vulcanization accelerator, is an important ingredient for vulcanized rubber (Xiang et al., 2015). In the vulcanization process, DPTT tends to react with sulfur ions or free radicals to accelerate the cross-linking reaction with rubber chains, thus shortening the vulcanization time and lowering the operating temperature. Inspired by this motivation, Xiang and colleagues introduced DPTT in the electrolyte for high-performance Li−S batteries (Xiang et al., 2019). As shown in Figure 7H, the soluble LiPSs can interact with DPTT. As a consequence, LiPSs can be rapidly converted into S_8_ and Li_2_S_2_ (Figure 7F). The UV-vis absorption spectra of LiPS solutions exhibit characteristic peaks at 310 and 420 nm which belong to S_6_^2−^ and S_4_^2−^, respectively. After the reaction with DPTT, the characteristic peak at 420 nm disappears and the peak at 310 nm weakens remarkably. In addition, a new peak at 265 nm emerges which can be assigned to S_2_^2−^. These evidences strongly suggest that the reaction between DPTT and LiPSs accelerates the sulfur conversion reactions. The battery with 4 wt% of DPTT exhibits excellent electrochemical performance that remains 914.7 mAh g^−1^ after 250 cycles at 0.5 C.

### Solid–Solid Transformation of LiPSs

The slow reaction kinetics results in the accumulation of LiPSs in the electrolyte, thus increasing the viscosity of the electrolyte and decreasing the sulfur utilization. To tackle these issues, a feasible strategy is to accelerate the formation of insoluble sulfides by adding redox mediators to react with soluble LiPSs. As a suitable case, the bis(4-nitrophenyl) carbonate (BNC) additive can interact with soluble LiPSs to form insoluble sulfides and 4-nitrophenol lithium. This approach can inhibit the shuttle effect and realize a stable electrochemical performance (Yang T. et al., 2019). Furthermore, to make a compromise between the effective immobilization of LiPSs and the rapid diffusion of Li^+^, Chen's group converted the soluble LiPSs into the solid organic LiPSs through the nucleophilic substitution reaction between 2,5-dichloro-1,4-benzoquinone (DCBQ) and LiPSs (Chen et al., 2021). They proved that the addition of DCBQ in the electrolyte can also effectively inhibit the shuttle effect. Specifically, the benzoquinone group in DCBQ facilitates the transfer of Li^+^ and reaction kinetics (Figure 7I). According to DFT simulations, the detailed reaction mechanism of the benzoquinone group on promoting the conversion of LiPSs was investigated, as shown in Figure 7J. In the first transition state (TS1), an S atom at the end of Li_2_S_4_ is close to the adjacent C atom of quinone groups. In the TS2 state, the S-S bond is broken and then bonded with adjacent C atoms. The calculated energy barrier for the TS2 state is 1.24 eV. In contrast, a higher energy barrier of 1.68 eV is reported for the S−S bond on benzene rings. These results indicate the effective promotion of the conversion of LiPSs by quinone groups in DCBQ.

### Formation of CEI Layer

From the perspective of the Li anode, the LiPS shuttle can be relieved by constructing a protective layer on its surface, namely solid electrolyte interface (SEI) (Bell et al., 2018). A stable SEI layer is also beneficial to maintain a flat surface on the Li anode and improve the diffusion rate of Li^+^ at the interface (Liu et al., 2019). The SEI counterpart in the positive electrode is the cathode electrolyte interface (CEI) which builds a barrier to hinder the diffusion of LiPSs (Kensy et al., 2021). Despite the importance, by far, there has been only limited research about CEI. Recently, Qian et al. put forward a new organic electrocatalytic additive of 1,1,2,2-tetrafluorovinyl-2,2,3,3-methyl acrylate ether (TTE) in the electrolyte to design advanced CEI layer on S/C composite cathodes (Qian et al., 2018). The *in-situ* formed CEI passive film was demonstrated to efficiently inhibit the diffusion of LiPSs from the cathode, and promote the formation of a dense, smooth, and uniform SEI layer on the anode, thereby suppressing the corrosion of the anode Li sheet.

## Conclusion and Outlook

In this review, we provide a systematic overview of organic electrocatalytic materials in Li−S batteries. The rational design of these catalysts is divided into different categories, according to their functionality, and we further make an in-depth discussion on their merits in sulfur conversion reactions. Despite the achievements in addressing the problems of Li−S batteries, the remaining challenges for elevating the overall performance need more attention. To further improve the reaction kinetics and promote the sulfur conversion efficiency as well as to clarify the regulation mechanisms by electrocatalysts, a few possible directions can be proposed that have yet attracted deserved attention but are worthy of in-depth exploration owing to their great perspectives.

### Material Optimization

Considering that the solid–liquid–solid transformations occur in the sulfur redox reactions, the electron and ion transfer channels may be blocked by insoluble sediments that will cover the surface of electrocatalysts, leading to the deactivation of active species and greatly deteriorated catalytic performance. In the future design, porous conductive frameworks with large specific surface area, such as 3D carbon fibers and highly conductive MOFs, can be introduced as matrixes of organic electrocatalysts. By employing these organic–inorganic composite catalysts, the obtained large electrolyte/matrix interfaces will induce rapid LiPS capture and even deposition of S and Li_2_S_2_/Li_2_S. Conducting substrates serve as excellent electrochemical reaction platforms for accelerating the mass and carrier transfers. This tactic is also helpful to immobilize small organic molecule catalysts, avoiding their dissolution. Of special notes is the way of their combinations, mainly including noncovalent π–π conjugation, covalent bond, Van der Waals force, and hydrogen bond. Screening effects can be carried out at the material design stage. Meanwhile, the effects of the active site configurations on catalysts should also be carefully checked. The key point is the precise regulation of the amount and spatial distribution of them. Besides, scientists should also note the coordination between different types of functional groups. The synergistic strategy may facilitate specific procedures of dynamic evolutions of sulfur species (depicted in Scheme 2).

Functional groups on the surface of organic electrocatalysts also determine their unique properties (Zhang A. et al., 2018). The surface wettability of organic electrocatalytic materials, such as bionic design in energy materials, can take effect on the cohesion with electrolyte that can be used to reduce the dosage of electrolyte and regulate the concentration gradient of LiPSs, avoiding the interference of other rate-determining steps. Moreover, owing to the aging characteristic of organic materials, the study on electrochemical/chemical/thermal stabilities of organic electrocatalysts under the battery operating conditions is an urgency to maintain their high activities.

### Mechanism of the Electrocatalytic Process

The catalysts for traditional Li−S batteries are mainly inorganic materials such as metal oxides and nitrides. There are many crystal planes exposed on their surfaces, and a variety of intermediate products may be generated. Therefore, it is difficult to distinguish the sites that act as catalysts. In the foreword, we mentioned that the effect of organic electrocatalytic materials on LiPSs mainly involves six steps of dynamic evolutions (Zhang M. et al., 2019). At present, the adsorption process merely is relatively clear. Organic materials mainly interact with Li cations or polysulfide anions to form adsorption clusters by the Lewis acidic–basic interaction and/or π–π conjugation. This phenomenon can be confirmed by the visible adsorption experiment of LiPSs and the peak position shifts of the corresponding XPS binding energies. However, most reports on organic electrocatalysis are narrated by macroscopic phenomena. Generally, indirect evidences are used to indicate the occurrence of catalytic processes, such as the increased CV integral area of symmetrical cell, the decrease of polarized voltage of charge/discharge platform, the XPS peak position shifts of specific elements, and the improvement of cell rate and cycling performance. Up to now, the specific processes of sulfur conversion by catalysts are yet clear and only few researchers pay attention to the crux of the matter.

**FIGURE 8:**
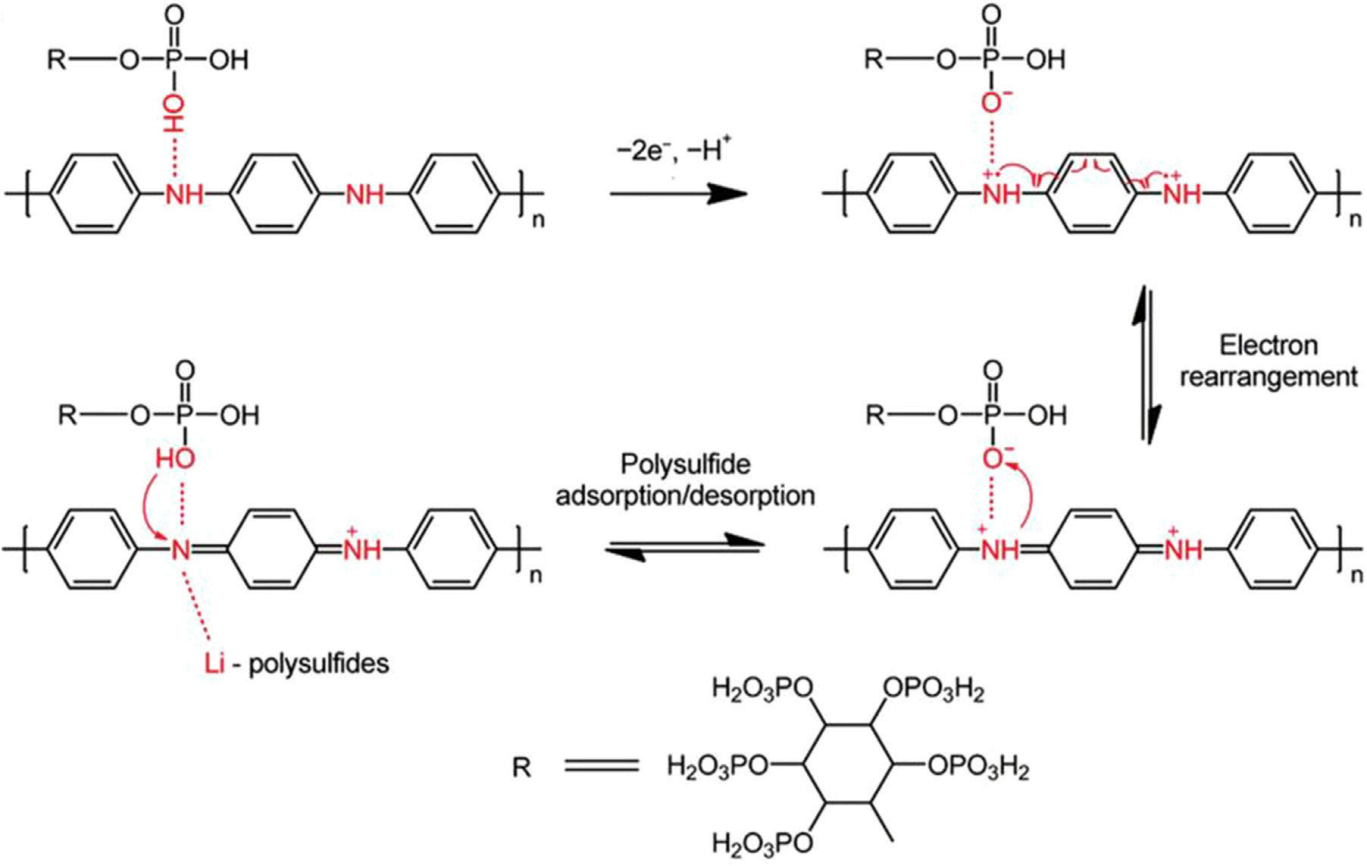
Schematic illustration of the stabilization and enrichment of quinonoid imines by PA and their reversible transformations in Li−S batteries. Figure reproduced from the data in Chen C.-Y. et al. (2017).

For example, Chen et al. pointed out that the lone pair electrons of N atoms in pyridine and pyrrole rings can be used as Lewis basic sites for Li^+^ adsorption (Chen C.-Y. et al., 2017). After the completion of adsorption, the change of nitrogen-localized environment will cause a great change in its adsorption energy. The adsorption energies toward the Li_2_S_8_ molecule increase from −1.25 and −1.28 eV of aniline nitrogen (-NH- and -NH^+^ = ) to −1.83 and −1.61 eV of quinone imine (-N = and -NH^+^). Accordingly, they proposed to use quinonid imine as an organic redox mediator to interact with LiPSs. It was found that, in this process, the amino functional groups in quinonid imine would undergo a reversible transition between the proton state (-NH^+^ = ) and the de-proton state (-N = ) under the action of phytic acid (PA) (Figure 8). In the following research, they polymerized benzene-1,4-dicarboxaldehyde with pyrrole to prepare an electrocatalyst based on porphyrin framework (POF) for sulfur conversion. According to theoretical simulations by different charge density analyses, they found the charge density increases significantly after the interaction between Li atoms in LiPSs and N atoms in POF, which suggests a strong electronic interaction between Li and N, forming a Li bond. The formation of Li bond will weaken Li−S binding energy and promote the decomposition of LiPSs. Owing to the high polarity and the conjugated structure of POF together with the existence of intermolecular polarization, the interaction between POF and sulfur species becomes stronger. The electronic density of states (DOS) shows that the 2p orbital of N in POF overlaps with the 3p orbital of S in LiPSs, implying the easy transfer of electrons. The conjugated polar POF affords delocalized electrons and appropriate electronic structures are accounted for the two essential advantages for the superior LiPSs catalysis.

Although in specific systems, some catalytic processes have been studied by means of experiments and theoretical calculations, the research on mechanisms of organic electrocatalytic materials in dynamic evolutions of sulfur species is still insufficient. More works should be conducted on follows:

1) Clarifying the catalytic path. It mainly involves the regulation and characterization of intermediate products, that is, the existing forms of organic electrocatalysts in different states of sulfur species, as well as the binding modes of each evolution state.
2) Monitoring the electrocatalytic process. The regulatory mechanism needs to be further elucidated with new tools. Most of the organic compounds cannot keep stable under the high-energy electron beam of conventional electron microscopes (TEM/SEM). This requires us to use noninvasive characterization methods in the detection of transition states. Therefore, it is necessary to combine a variety of electrochemical *in-situ* characterization techniques [such as atomic-force-microscope (AFM), UV-vis, FTIR, Raman, and XPS spectroscopes].
3) Combining homogeneous and heterogeneous catalysis. Generally, organic electrocatalytic materials can interact with electrolyte solvent, as a typical case, most small organic molecular materials can be dissolved in electrolyte. Since soluble LiPSs mainly appear in electrolyte in the form of solvated molecules or clusters, if one end of the electrocatalytic material is fixed on the matrix and the other end is soaked in the solvent, homogeneous adsorption of LiPSs will occur in the part of the electrolyte, which greatly improves the adsorption and catalytic conversion efficiency and favors the uniform deposition of Li_2_S_2_/Li_2_S.

### Challenges of Practical Applications

The study on the activities of organic electrocatalytic materials under different working conditions is the basis of their practicality. Owing to the existence of ohmic impedance, the internal temperature will rise during continuous operation. In addition, the high reactivity of the Li metal in the anode can react with the electrolyte to release CO_2_, H_2_O, and other gases, resulting in the increase of internal pressure. Under these conditions, temperature, pressure, operation duration, and charge/discharge rate may cause the aging of organic materials, leading to the degradation of its structure integrity and function, or even inactivation. These easy neglectful factors need to be taken just as seriously.
